# Anti-Inflammatory and Antioxidant Properties of *Malapterurus electricus* Skin Fish Methanolic Extract in Arthritic Rats: Therapeutic and Protective Effects

**DOI:** 10.3390/md20100639

**Published:** 2022-10-14

**Authors:** Abeer H. Elmaidomy, Esraa M. Mohamed, Hanan F. Aly, Eman A. Younis, Shams Gamal Eldin Shams, Faisal H. Altemani, Mubarak A. Alzubaidi, Mohammed Almaghrabi, Adnan Al Harbi, Faisal Alsenani, Ahmed M. Sayed, Usama Ramadan Abdelmohsen

**Affiliations:** 1 Department of Pharmacognosy, Faculty of Pharmacy, Beni-Suef University, Beni-Suef 62511, Egypt; 2 Department of Pharmacognosy, Faculty of Pharmacy, MUST, Giza 12566, Egypt; 3 Department of Therapeutic Chemistry, Pharmaceutical and Drug Industries Research Institute, National Research Centre, El Bouhouth St., Dokki, Giza 12622, Egypt; 4 Department of Medical Laboratory Technology, Faculty of Applied Medical Sciences, University of Tabuk, Tabuk 71491, Saudi Arabia; 5 Department of Biological Sciences, Faculty of Science, King Abdulaziz University, Jeddah 21589, Saudi Arabia; 6 Pharmacognosy and Pharmaceutical Chemistry Department, Faculty of Pharmacy, Taibah University, Al Madinah Al Munawarah 42353, Saudi Arabia; 7 Clinical Pharmacy Department, College of Pharmacy, Umm Al-Qura University, Makkah 21955, Saudi Arabia; 8 Department of Pharmacognosy, College of Pharmacy, Umm Al-Qura University, Makkah 21955, Saudi Arabia; 9 Department of Pharmacognosy, Faculty of Pharmacy, Nahda University, Beni-Suef 62513, Egypt; 10 Department of Pharmacognosy, Faculty of Pharmacy, Minia University, Minia 61519, Egypt; 11 Department of Pharmacognosy, Faculty of Pharmacy, Deraya University, 7 Universities Zone, New Minia 61111, Egypt

**Keywords:** *Malapterurus electricus*, anti-inflammatory, COX-2, molecular dynamics simulation

## Abstract

The protective and therapeutic anti-inflammatory and antioxidant potency of *Malapterurus electricus* (F. Malapteruridae) skin fish methanolic extract (FE) (300 mg/kg.b.wt/day for 7 days, orally) was tested in monosodium urate(MSU)-induced arthritic Wistar albino male rats’ joints. Serum uric acid, TNF-α, IL-1β, NF-𝜅B, MDA, GSH, catalase, SOD, and glutathione reductase levels were all measured. According to the findings, FE significantly reduced uric acid levels and ankle swelling in both protective and therapeutic groups. Furthermore, it has anti-inflammatory effects by downregulating inflammatory cytokines, primarily through decreased oxidative stress and increased antioxidant status. All the aforementioned lesions were significantly improved in protected and treated rats with FE, according to histopathological findings. iNOS immunostaining revealed that protected and treated arthritic rats with FE had weak positive immune-reactive cells. Phytochemical analysis revealed that FE was high in fatty and amino acids. The most abundant compounds were vaccenic (24.52%), 9-octadecenoic (11.66%), palmitic (34.66%), stearic acids (14.63%), glycine (0.813 mg/100 mg), and alanine (1.645 mg/100 mg). Extensive molecular modelling and dynamics simulation experiments revealed that compound **4** has the potential to target and inhibit COX isoforms with a higher affinity for COX-2. As a result, we contend that FE could be a promising protective and therapeutic option for arthritis, aiding in the prevention and progression of this chronic inflammatory disease.

## 1. Introduction

Gout is regarded as an inflammatory response caused by the deposition of monosodium-urate (MSU) crystals around the joints [1]. When it comes to the progression of gout, the inflammatory response comes first. The underlying process is that MSU-crystals cause intra-articular inflammation by stimulating complement organization and increasing macrophages and neutrophils, which accelerates synovial and cartilage tissue destruction and may progress to joint damage [2]. MSU-crystals recruit nucleotide-binding-oligomerization-domain-like-receptor-protein-3 (NLRP3) to the release of inflammatory mediators such as interleukin-6 (IL-6) and interleukin-1β (IL-1β) [3]. The high release of IL-1β is thought to be the first symptom of gout [4].

Redox signaling particles, such as reactive oxygen species (ROS), can mediate NLRP3 inflammasome production, thereby impeding NLRP3-mediated inflammatory responses [4]. The NLRP3 inflammasome becomes significant in various inflammatory disorders. Furthermore, its unusual activation has been linked to the pathogenesis of inflammatory disorders such as Alzheimer’s disease, obesity, multiple sclerosis, diabetic problems, inflammatory bowel syndromes, and gout [5]. Toll-like receptors (TLRs) are a type of recognition receptor found in the innate immune system. According to recent evidence, TLRs may be involved in the recognition and activation of MSU-crystals [6]. TLRs bind to Myeloid-differentiation-Factor88 (MyD88), resulting in a recruiting, complex, and inflammatory response. All of this has been discovered by stimulating the transformation of growth-factor- (TGF-β) kinase activity, which activates the transcription-factor-nuclear-factor-kappa-B (NF-B) and initiates the transcription and expression of the IL-1β-specialized prototype pro-IL-1β gene. Nonsteroidal anti-inflammatory drugs (NSAIDs) and colchicine are the primary clinical treatments for gouty arthritis. However, the side effects limit its clinical application [7]. To be clear, indomethacin may cause kidney toxicity in elderly patients. Furthermore, long-term colchicine therapy is expected to impair hematopoietic and bone marrow function [1].

Marine biodiversity has sparked considerable interest in allowing a massive hidden source of chemicals with numerous remedial and feed demands [8]. Among them are fish, which are widely consumed due to their high concentrations of polyunsaturated fatty acids (PUFA, or omega (ω)-fatty acids), protein, and essential amino acids [9]. For example, mackerel, sardines, anchovies, and some salmon species are high in polyunsaturated omega-3 fatty acids (ω-3 PUFA), which include 22:6 ω-3 (docosahexaenoic acid (DHA) and 20:5 ω-3 (eicosapentaenoic acid (EPA) (EPA). These essential fatty acids have numerous nutraceutical benefits related to blood clotting [10], inflammation [11], the central nervous system (CNS) [12], and the cardiovascular system (CVS) [13,14].

*Malapterurus electricus* (electric catfish), in particular, is found among rocks or roots in turbid and/or black waters with poor visibility as it prefers to stand or sluggish water, and is found primarily in western, central tropical Africa, and the Nile River [15]. *M. electricus* has a general body shape that has been described as a bloated sausage [16]. The head is slightly depressed, and the body can extend to 1220 mm in length. The eyes are small, with a rounded snout and thick lips [15]. *M. electricus* have greyish brown backs and sides that fade to off white or cream on the ventral surfaces of the head and body. The sides of the body have black spots. *M. electricus* is consumed as a meal in parts of Africa and is occasionally found in the pet trade as an aquarium fish [17]. *M. electricus’* electric organs have been used in studies of axonal transport, neuronal metabolism, and transmitted discharge [18,19].

Previous research has found that unsaturated fatty acids (UFA) and amino acids play roles in anti-inflammatories by directing cell migration and proliferation, phagocytic capability, and the management of inflammatory indicators [20]. Thus, the current study investigated the chemical composition of *M. electricus* fish skin crude extract and determined its potential as an anti-inflammatory and antioxidant agent in the inflammatory joint of male-Wistar-rats caused by MSU-crystals, using in silico studies to identify the most likely mechanisms-of-action.

## 2. Results

### 2.1. Effects of Fish Extract on Ankle Swelling in MSU Crystal-Gouty Arthritis Rats

Uric acid levels were found to be significantly higher in MUS-induced arthritic rats (positive group) than in the control group with percentage increase reached to 263.33%. In the protective group, the percentage of reduction in uric acid level reached to 54.13%. However, arthritic rats treated with FE showed a gradual reduction in uric acid levels, reaching 4.00 ± 0.90 mg/dL with percentage reduction 63.30%, after 7 days of treatment. Indomethacin reduced the uric acid level from 3.50 ± 0.60 mg/dL with percentage reduction 67.89% (see Table 1). 

Furthermore, there were no variations in the ankle swelling level at baseline. MSU-crystal increased ankle swelling levels in MSU-induced arthritis compared to control group (0.500 ± 0.03 mm) after 24 h. While the ankle swelling diameter reached to 6.55 ± 0.56 mm post 7 days of MSU injection. The Ankle swelling in the protective group was 1.32 ± 0.44 mm after 24 h and 1.90 ± 0.9 mm after 7 days. However, arthritic rats treated with FE showed a gradual reduction in ankle swelling from 1.95 ± 0.66 mm after 24 h to 1.89 ± 0.11 mm after 7 days of treatment. Indomethacin reduced ankle swelling diameter from 1.00 ± 0.43 mm after 24 h to 1.80 ± 0.20 mm after 7 days (Table 1).

### 2.2. Effects of Fish Extract on Inflammatory Markers TNF-α, IL-1β, and NF-𝜅B in Synovial Tissues Homogenate

Significant increases in all inflammatory markers in MUS-induced arthritis in rats compared to controls, with percentage increases of 678.64, 982.62, and 203.03% for TNF -α, IL-1β and NF-𝜅B, respectively. Significantly lower levels of inflammatory markers were observed in the MUS- protected group with percentages of reduction reached to 51.43, 53.16 and 50.00 %, respectively for TNF -α, IL-1β and NF-𝜅 compared to the MUS- group. However, TNF -α, IL-1β and NF-𝜅B recorded percentages of reduction amounted 71.43, 73.68 and 60.00%, respectively, in arthritic treated group with FE (Table 2).

### 2.3. Effects of Fish Extract on Oxidative Stress and Antioxidant Markers in Synovial Tissues Homogenate

GSH reductase, catalase, GSH, and SOD levels were significantly reduced in arthritic rats (50.00, 55.56, 62.95, and 58.82%, respectively) in Table 3. While a significant increase in MDA level (173.17%) was found. GSH reductase, catalase, GSH, MDA, and SOD levels in the arthritic-protected group with FE changed by 37.5, 75.00, 70.96, 30.36, and 60.00%, respectively. Furthermore, GSH reductase, catalase, GSH, MDA, and SOD levels in arthritic-treated rats treated with FE improved by 97.50, 100.00, 145.95, 54.11, and 80.95%, respectively, when compared to standard drug (60.00, 100.00, 139.95, 51.10, and 79.14%, respectively). 

### 2.4. Histopathological Parameters 

#### 2.4.1. Histopathological Findings

**Figure 1:**
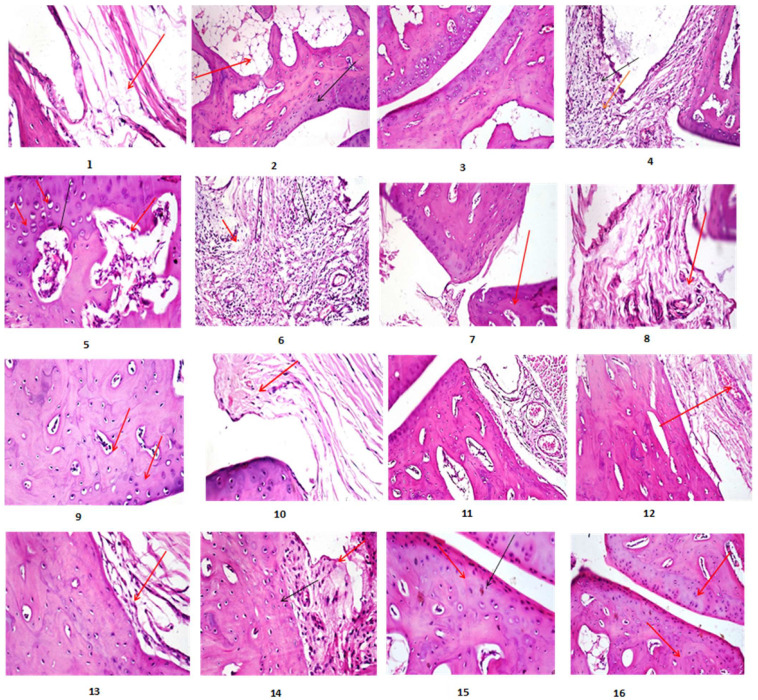
Histopathological alterations of Arthritic and treated rats. Photomicrograph (1), of control rats showed synovial membrane showing normal histological structure (arrow) (H&EX400), Photomicrograph (2) of control rats showed articular surface showing normal histological structure (arrow) (H&EX200), Photomicrograph (3) of control rats showed showing normal histological structure (H&EX400), Photomicrograph (4) of Arthritic rats showed synovial membrane with heavy infiltration of inflammatory cells (arrow) (H&EX200), Photomicrograph (5) of Arthritic rats showed articular surface showing irregularity of articular surface (arrow) with widening of trabecular space and necrosis (H&EX400), Photomicrograph (6) of Arthritic rats showed synovial membrane showing heavy infiltration of inflammatory cells (arrow) (H&EX400), Photomicrograph (7) of Arthritic rats treated with FE showed articular surface with smooth articular surface (arrow) with normal trabecular space and few necrosed chondrocytes (H&EX400), Photomicrograph (8) of Arthritic rats treated with FE showed synovial membrane with few inflammatory cells and mild edema (arrow) (H&EX200), Photomicrograph (9) of Arthritic rats treated with FE showed articular surface with smooth articular surface (arrow) with normal trabecular space and few necrosed chondrocytes (H&EX200), Photomicrograph (10), of Arthritic rats treated with FE showed synovial membrane with mild edema (H&EX400), Photomicrograph (11) of Arthritic-protective rats articular surface showing nearly normal and smooth articular surface with mild edema of synovial membrane (H&EX200), Photomicrograph (12) of Arthritic-protective rats showed synovial membrane showing mild edema and congestion of synovial membrane (H&EX200). Photomicrograph (13), of Arthritic -protective rats’ articular surface showing normal articular surface and synovial membrane (H&EX400). Photomicrograph (14), of Arthritic -protective rats showing normal articular surface and synovial membrane (H&EX400). Photomicrograph (15) of Arthritic rats treated with standard drug showing smooth articular surface (arrow) with normal trabecular space and few necrosed chondrocytes (H&EX400). Photomicrograph (16) of Arthritic rats treated with standard drug showed, articular surface with normal and smooth articular surface and few necrosed chondrocytes (H&EX400).

Arthritic rats (G2, Photomicrographs 4–6, Figure 1) showed articular surface irregularity and deformity with necrosis of a significant number of chondrocytes, synovial membrane and subcut tissue showed heavy infiltration of inflammatory cells with edoema and congestion of blood vessels, and bone trabeculae showed widening of trabecular space compared to control (Photomicrographs 1–3, Figure 1). All of the aforementioned lesions were significantly improved in arthritic rats treated with both FE and a standard drug (G4, G5, Photomicrographs 7–10, 15,16, Figure 1). The articular surface and synovial membrane of protective groups injected with MSU and co-administered with FE (G3, Photomicrographs 11–14, Figure 1) showed nearly normal structure.

#### 2.4.2. Lesion Score (Scoring of Histopathological Alterations of All Treated Groups)

**Figure 2:**
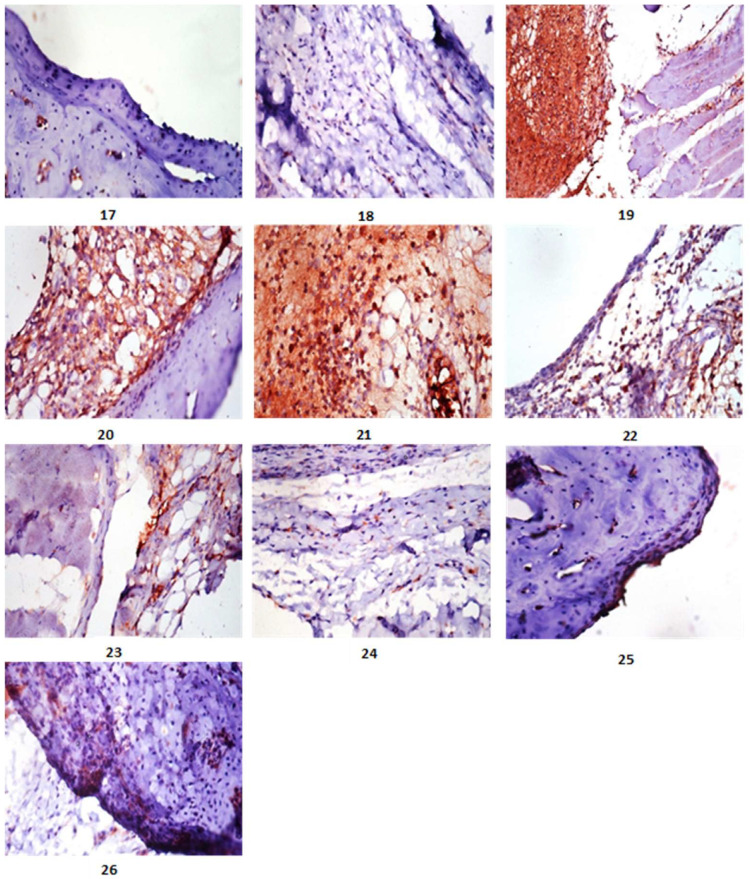
Expression of iNOS in Arthritic rats and different treated groups. Photomicrograph (17) of immunostaining of iNOS of control rats showed articular surface with no immune reactive cells (iNOS X400). Photomicrograph (18): of immunostaining of iNOS of control rats showed articular surface showing no immune reactive cells (iNOS X400). Photomicrograph (19) of immunostaining of iNOS of Arthritic rats showed synovial membrane with strong immune expression of iNOS (iNOS X400). Photomicrograph (20): of immunostaining of iNOS, of Arthritic synovial membrane with strong immune expression of iNOS (iNOS X400). Photomicroograph (21) of immunostaining of iNOS of Arthritic rats showed synovial membrane showing strong immune expression of iNOS (iNOS X400). Photomicrograph (22) of immunostaining of iNOS of Arthritic rats treated with FE showed articular surface and synovial membrane with weak immune expression of iNOS (iNOS X400). Photomicrograph (23): of immunostaining of iNOS of Arthirtic rats treated with FE showed synovial membrane with weak immune expression of iNOS (iNOS X400). Photomicrograph (24): of immunostaining of iNOS, of Arthritic rat’s protective with FE showed synovial membrane with weak immune expression of iNOS (iNOS X400). Photomicrograph (25): of immunostaining of iNOS, articular surface of Arthritic–protective rats showing very weak immune expression of iNOS (iNOS X400). Photomicrograph (26): of immunostaining of iNOS, articular surface of Arthritic rats treated with standard drug showing very weak immune expression of iNOS (iNOS X400). iNOS quantitative measurement is indicated by the percentage of immune reactive cells stained in the articular surface and synovial membrane of arthritic, protected and treated rats.

All lesions found in bone tissue were graded based on their severity. Table 4 shows a significant reduction in score lesion in either the treated or protective groups when compared to the Arthritic group. In standard drug-treated Arthritic rats, similar results were obtained. Table 5 also showed a significant reduction in the percentage expression of iNOS in the treated and protected groups when compared to the Arthritic group. The immunostaining expression of iNOS% area in different treated groups. iNOS immunostaining revealed no immune-reactive cells in the control group (Photomicrographs 17, 18; Figure 2). Sections from arthritic rats (G2, Photomicrographs 19–21; Figure 2) revealed a high level of iNOS expression. In contrast, arthritic rats treated with FE as well as standard drug (G4, G5) showed weak positive immune-reactive cells (Photomicrographs 22, 23, and 26; Figure 2) with percentages of iNOS reduction amounted to 31.87 and 48.46 in articular-surface and synovial membrane and for group 4 respectively. While the percentages of iNOS reduction for the articular surface, synovial membrane, and group 5 were 24.57 and 43.47%, respectively. However, the protective group (G3, Photomicrographs 24, 25; Figure 2) showed very few to no positive immune-reactive cells, with iNOS reduction percentages of 31.87 and 48.46% for the articular surface and synovial membrane, respectively.

### 2.5. Phyto-Chemical Investigation of Malapterurus electricus Crude Extract 

#### 2.5.1. GC-MS Analysis

*M. electricus* skin fish provided 10% *v*/*w* oil fresh weight, which has a faint yellow- color, odorless, lighter than water, and picks up a heavy white turbidity when left in the chamber. The GC-MS analysis revealed that 8 compounds accounted for 97.91% of all detected peaks (Table 6, Figure S1). These identified compounds belong to the fatty-acid chemical class Table 6, which includes saturated-fatty-acids (2 SFA, 49.29%), mono-unsaturated-fatty-acids (3 MUFA, 37.88%), and poly-unsaturated-fatty-acids (3 PUFA 10.74%).

The total unsaturated fatty acid (UFA) and SFA content were nearly identical, accounting for 48.62 and 49.29%, respectively. Among UFA, vaccenic and 9-octadecenoic acids were the most generous MUFA, accounting for approximately 24.52% and 11.66% of total MUFA, respectively. Combined *n*-3 PUFA (C20:4, C20:5, and C22:6) accounted for 10.74% of total FA in crude *M. electricus* fish oil, with arachidonic, 5.8,11,14,17-eicosapentaenoic, and 4,7,10,13,16,19-docosahexaenoic acids accounting for 3.41, 1.33, and 6.00%, respectively. Palmitic and stearic acids were the most abundant SFA, accounting for 34.66% and 14.63% of total FA, respectively (Table 6).

#### 2.5.2. Physicochemical Investigation of *Malapterurus electricus* Crude Extract

**Figure 3:**
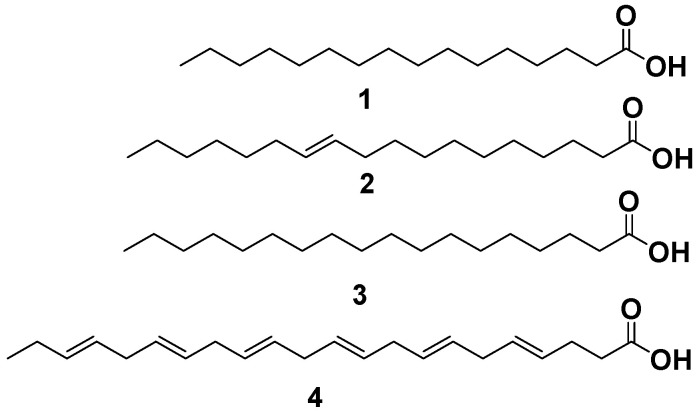
Structures of compounds isolated from *M. electricus* extract. **1**, palmitic acid; **2**, vaccenic acid; **3**, stearic acid; and **4**, 4,7,10,13,16,19-docosahexaenoic acid.

Physicochemical and chromatographic properties, spectral investigations (UV, 1H, DEPT-Q NMR, and mass), as well as correlations with early papers and some standards, established that *M. electricus* fish skin crude extract provided the following recognized compounds: **1**, palmitic acid [8]; **2**, vaccenic acid [8]. Furthermore, **3**, stearic acid [8] and **4**, 4,7,10,13,16,19-docosahexaenoic acid [8] (Figure 3 and Figures S2–S9).

#### 2.5.3. Amino Acid Content

Amino acids are required for the synthesis of a wide range of proteins, including carriers of CO_2_, oxygen, structural proteins, and vitamins [21]. Table 7 and Figure S10 show the amino acid content of *M. electricus* extract. The total amino acid content of the *M. electricus* extract was 4.584 mg/100 mg, with a high concentration of glycine (0.813 mg/100 mg) and alanine (1.645 mg/100 mg). *M. electricus* extract contained 1.213 mg/100 mg of essential amino acids such as threonine, valine, isoleucine, leucine, phenylalanine, histidine, and lysine (0.144, 0.206, 0.108, 0.247, 0.124, 0.142, and 0.242 mg/100 mg, respectively).

### 2.6. Molecular Modeling Study

Fatty acids, particularly unsaturated ones, have been shown to have significant anti-inflammatory potential [22,23,24]. The COX pathway, along with its main product prostaglandin E2 (PGE-2) [25,26], is one of the primary inflammatory mediators implicated in the inflammatory phase of joints and its accompanying pain. It was recently discovered that inhibiting COX-2 specifically was associated with faster pain relief and decreased joint inflammation in experimental mice [27]. Based on their structural similarity to arachidonic acid (AA), the primary precursor of the COX enzyme, we investigated the isolated fatty acids’ potential interactions with both COX-1 and COX-2. Each fatty acid’s modelled structure was docked into the active sites of both COX-1 and COX-2 (PDP codes: 3KK6 and 3HS5, respectively). The convergent scores for all docked compounds (**1**–**4**) ranged from −7.34 to −8.75 kcal/mol. 

To gain a better understanding of each isolated fatty acid’s affinity for the active sites of COX-1 and COX-2, the best generated docking pose for each was subjected to MDS-based ΔGbinding calculation using the Free Energy Perturbation method (FEP) [28]. As a result, compound **4** was found to have the highest affinity for both enzymes (COX-1 and COX-2) with a slightly higher affinity for COX-2 (Table 8). Compound **4** has the most double bonds of any compound and thus has the least flexible molecules. As a result of compound **4′**s low flexibility in comparison to the remaining fatty acids, its entropic component in the binding free energy was relatively significant, and thus its affinity towards the enzymes’ active site was the best (Table 8). 

**Figure 4:**
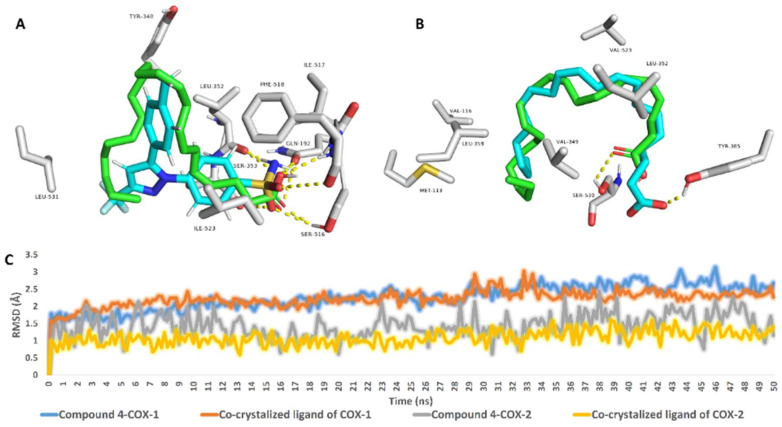
Docking poses of 4,7,10,13,16,19-docosahexaenoic acid (compound **4**) (green-colored structure) inside the active sites of both COX-1 and COX-2 aligned with that of the co-crystalized ligands (cyan-colored structures) (**A** and **B**, respectively). RMSDs of compound **4** and the co-crystalized ligands inside the active sites of both COX-1 and COX-2 over 50 ns of MDS (**C**).

According to this preliminary finding, the interactions of compound **4** inside the active sites of both enzymes were investigated further using 50 ns MDS experiments. Before beginning the simulations, the structural alignment of compound **4** and the co-crystalized ligands inside the active sites of COX-1 and COX-2 revealed perfect matches in terms of binding orientation and interactions (Figure 4). In the case of COX-1, both compound **4** and the co-crystalized ligand exhibited the same hydrophobic and hydrophilic interactions (Figure 4A). Except for H-bonding, compound **4** established a single H-bond with SER-530, whereas the shorter co-crystalized fatty acid (i.e., arachidonic acid) established a single H-bond with TYR-385 (Figure 4B). Over the 50 ns MDS, compound **4** and each co-crystalized ligand inside the active site of both enzymes (i.e., COX-1 and COX-2) demonstrated nearly identical binding stability with average RMSDs of 2.2 for COX-1 and 1.3 for COX-2 (Figure 4C). 

**Figure 5:**
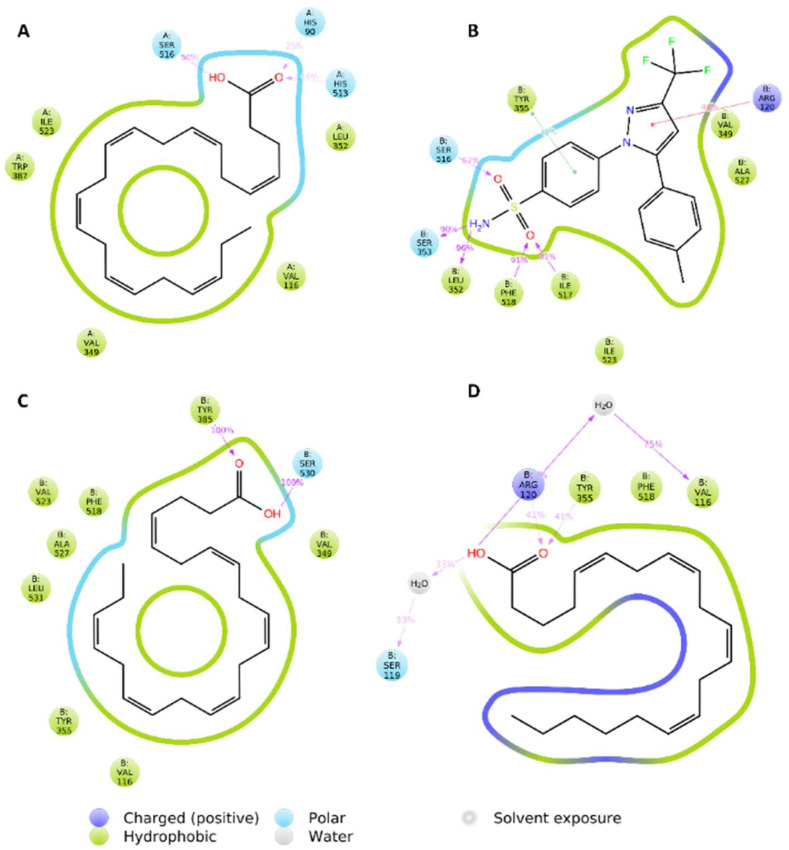
Protein-ligand contacts of 4,7,10,13,16,19-docosahexaenoic acid (compound **4**) (**A**,**C**) and the co-crystalized ligands (**B**,**D**) inside the COX-1′ and COX-2′s binding sites over 50 ns of MDS.

Over the course of simulation, the most populated binding poses for compound **4** and both co-crystalized ligands demonstrated dynamic binding behavior of compound **4** inside the active sites of both enzymes. Compound **4′**s fatty tail established stable hydrophobic interactions with VAL-116, VAL-349, LEU-352, TRP-387, and ILE-523, as shown in Figure 5A. Furthermore, the carboxylate moiety formed three H-bonds with HIS-90, HIS-513, and SER-516. It is worth noting that, with the exception of the stable H-bond with SER-516, the binding mode of the co-crystalized ligand of COX-1 changed over the course of simulation and became different from that of compound **4** (Figure 5B). Similarly, within COX-2′s active site, compound **4**′ long hydrocarbon chain established multiple hydrophobic interactions with 6 amino acids (VAL-116, VAL-349, TYR-355, PHE-518, ALA-527, and LEU-531), whereas arachidonic acid (COX-2′s co-crystalized fatty acid) interacted with only three (i.e., VAL-116, TYR-355, and PHE-518). As a result, compound **4′**s significant hydrophobic interactions within the active site of COX-2 explain its higher affinity in terms of Gbinding over arachidonic acid (Table 8). In terms of H-bonding, compound **4** formed very stable ones with SER-530 and TYR-385, whereas arachidonic acid formed fewer stable ones with ARD-120 and TYR-355, as well as a single water bridge with SER-119 (Figure 5C,D). 

## 3. Discussion

Gout is an auto-inflammatory condition associated with elevated blood urate levels due to the deposition of MSU-crystals in and around joints [29], which resulted in an increase in neutrophils. Neutrophils correlate with the elevation of IL-1β, IL-8, and TNF-α levels, which are necessary cytokines in inflammation and significant mediators implicated in the pathogenesis of gout [2]. A network that stimulates the interpretation of NF-κB regulates the expression of several cytokines. NF-κB is a key regulator of proinflammatory gene expression that can increase the expression of a variety of cytokines, including TNF-α, IL-1β, and IL-8 [30]. Many inflammatory genes, including cytokines, adhesion molecules, and chemokines, are activated by NF-κB [31]. As a result, downregulation of the NF-𝜅B signaling pathway may be an appropriate approach for gout treatment. In the current study, we measured the levels of TNF-α, IL-1β, and NF-κB in arthritic rats caused by MUS in a rat model [32,33,34,35], while management or protection by fish extract reduced the levels in the serum.

Furthermore, another inflammatory process associated with gout is oxidative stress. The current findings revealed a significant increase in MDA levels, as well as a distinct decrease in catalase, GSH reductase, GSH, and SOD. This oxidative state is caused by the production of ROS and pro-inflammatory cytokines [36], which results in hyaluronic acid depolymerization, degradation of proteoglycans and collagen, protein decomposition, and inhibition of proliferation. These cells are known to have low levels of antioxidants such as catalase and SOD [37]. In the state of gout, crystals increase the production of NO [38], and when it combines with O_2_, peroxy nitrite (ONOO) is formed, disrupting cellular proliferation, connective material degradation, and joint degeneration [39]. NO mediates the body’s innate immune response. Extracellular catalysts can initiate iNOS expression by activating NO signaling pathways [40,41,42,43,44]. In the case of arthritis, NO now interferes with inflammation [40]. Similarly, as shown in our findings, gouty arthritis synovial tissue has elevated iNOS interpretation (Figure 2). Furthermore, chondrocyte apoptosis occurs as a result of the activities of reactive nitrogen intermediaries and proinflammatory cytokines, which contribute to significant cartilage loss. Bone destruction is accelerated by the release of cytokines such as GM-CSF and the activation of NF-κB, which promotes osteoclast differentiation and cellular invasion of the cartilage-affected surface [42]. TNF-α, IL-1β, IL-6β, and GM-CSF can promote the management of adhesion molecules, other inflammatory cytokines, and chondrocyte and osteoclast activation, all of which contribute to joint loss [44].

The present study investigated the GC/MS composition, amino acids content, as well as the phytochemical composition of FE and evaluated its anti-inflammatory and antioxidant potency in treatment or protective arthritic Wistar albino male rats’ joints. The phytochemical analysis revealed that FE was high in fatty acids (both saturated and unsaturated) and amino acids (essential, non-essential). The most abundant compounds were vaccenic (24.52%), 9-octadecenoic (11.66%), palmitic (34.66%), stearic acids (14.63%), glycine (0.813 mg/100 mg), and alanine (1.645 mg/100 mg). In addition, the current findings stated that either treatment or protective—arthritic inflamed rats with FE showed significant improvement in all mentioned parameters; additionally, the articular surface and synovial membrane showed nearly normal structure with low lesion score in irregularity and deformities of the articular surface, necrosis of chondrocytes and synovial membrane infiltrated with inflammatory cells, and widening of the trabecular space (Table 1, Table 2, Table 3, Table 4 and Table 5, Figure 1 and Figure 2). Furthermore, there is little to no positive iNOS expression in inflamed immune-reactive cells. Furthermore, cytokine modulation was associated with improvements in inflammatory, clinical, and histological frameworks in animals treated with FE, indicating that FE is a promising treatment for arthritis. Overall, these findings are significant because, while anti-arthritic medications reduce inflammation, they do not consistently prevent or improve bone erosion and cartilage damage. The reduction of inflammatory infiltrates in the cartilage of animals treated with FE may be related to the modulatory activity of these inflammatory mediators, including the downregulation of TNF-α, IL-1, 2, 6, and GM-CSF, the reduction of NO, and the increase of IL-10, indicating that FE reduced inflammatory infiltrate, synovial hyperplasia, and bone erosion [20].

The current study’s findings agreed with those of Bahadori and colleagues, et al., in their 2010 report [45], which published the value of fish extract or *n*-3 PUFA in rheumatoid arthritis. These findings were consistent with those of Ruggiero et al. 2009 [46], who described more reliable data supporting the efficacy of omega-3 PUFAs in pain relief and the treatment of nonsteroidal anti-inflammatory drugs. Alam et al., 1993 [47] investigated the effects of dietary fats on inflammatory mediators in alveolar bone. They discovered that these dietary lipids had a significant impact on the formation of fatty acids in bone lipids. Arachidonic-acid accumulations in the overall phospholipids of the mandibles and maxillae were significantly lower in rats fed fish oil diets. Arachidonic acid is a precursor to prostaglandin-E2 and leukotriene C4. A significant decrease in its concentration could result in lower levels of eicosanoids in the alveolar bone. Furthermore, early research established that omega-3 fatty-acid-derived lipid-mediators, recognized as repairing and protecting, may play an important role in repairing inflammation due to their strong anti-inflammatory activities. These mediators suppress inflammation even further, cause bone resorption, and directly influence osteoclast differentiation [48,49,50]. Several studies have found that n-3 PUFA eicosapentaenoic acid (EPA) and docosahexaenoic acid (DHA) rich in fatty fish oils have significant anti-inflammatory properties and beneficial effects on bone metabolism, most likely by inhibiting proinflammatory mediators such as prostaglandin E2, IL-1, 6, and TNF-α [50,51].

Furthermore, Saxena et al., 1984 [52] stated that amino acids (l-phenylalanine, dl-isoleucine, l-isoleucine, and l-leucine) had anti-inflammatory action by interfering with prostaglandin synthesis and/or action. Furthermore, Lee et al., 2017 [53] discovered that leucine, valine, and isoleucine inhibited NO production as well as inducible nitric oxide synthase mRNA expression. Glutamine has been shown to have an anti-inflammatory effect by inhibiting pro-inflammatory chemokine and cytokine production, which was achieved through excessive glutamine nutrient absorption, leading to the strengthening elimination of the inhibitor of nuclear-factor-kappa-B-kinase (Iκκ) action and the reduction of the inhibitor of nuclear-factor-kappa-B-kinase subunit-beta (IκB) degradation [54]. Glutamine is also known to inhibit NF-κB and P38 mito-gen-activated-protein-kinase (MAPK) pathway signaling factors [54,55]. Arginine has anti-inflammatory properties such as glutamine in that it inhibits chemokine re-ply of IL-8 production and inhibits Iκκ action [54,55]. Cysteine has been shown to inhibit IL-8 induced by TNF-α [56]. Cysteine inhibits NF-κB activation even further when combined with TNF-α [56]. Kynurenic acid is a byproduct of tryptophan metabolism [57]. Kynurenic acid can reduce IL-6 and TNF-α levels [58]. Furthermore, He et al., 2018 and Liu et al., 2017 [59,60] stated that the functions of amino acids (essential and non-essential) in inflammation are primarily associated with overcoming oxidative stress and inhibiting proinflammatory cytokines the expression. MAPK, NF-B, Nrf2, ACE2, iNOS, mTOR, CaSR, and GCN2 are among the signaling processes that carry out these functions.

Consequently, Omega-3 fatty acids and protein-rich FE have been shown to reduce inflammation which could be attributed to inhibitory and modulatory actions on the production and release of nitric oxide and cytokines, both of which are involved in disease pathogenesis. According to molecular modelling and dynamics simulation, one of the major components of the crude extract (compound **4**) has the potential to target and inhibit COX isoforms with a higher affinity for COX-2. 

## 4. Materials and Methods

### 4.1. Fish Collection

*M. electricus* fish-were-purchased-from-a-local-market in Beni-Suef, Egypt, in May 2022, and then identified using a fish-identification-key [61]. A voucher specimen (2022-BuPD-87) was archived-at Beni-Suef University’s Department-of-Pharmacognosy, Faculty of Pharmacy. Following purchase, the fish was-stored in plastic-bags and-preserved with dry ice.

### 4.2. Chemicals and Reagents

El-Nasr-Company for Pharmaceuticals-and Chemicals, Cairo, Egypt, supplied methanol (MeOH), dichloromethane (DCM), *n*-hexane-(*n*-hex., -boiling point b.p. 60–80 °C), ethyl acetate—(EtOAC), sulphuric-acid, and sodium-bicarbonate. Deuterated solvents, such as chloroform (CDCl_3_) and dimethyl sulfoxide (DMSO-d6), were provided by Sigma-Aldrich for spectroscopic analyses (Saint-Louis, MO, USA).

Column chromatography- (CC) was performed using silica-gel 60 (63–200 m, E. Merck, Sigma-Aldrich), and vacuum liquid chromatography was performed using silica gel G F254 (El-Nasr-Company for Pharmaceuticals-and Chemicals, Egypt) (VLC). TLC was carried out on pre-coated silica gel 60-GF254 plates (E. Merck, Darmstadt, Germany; 20 20 cm, 0.25 mm thick). After spraying with para-anisaldehyde—(PAA)—reagent, spots were visualized by heating at 110 °C (85:5:10:0.5 absolute EtOH: sulfuric acid: glacial acetic acid: PAA). Furthermore, Biosystems-SA Costa Brava-30, Barcelona (Spain) and DiaSys Diagnostic Systems-GmbH, Holzheim, Germany—purchased all the kits-used in the biological study.

### 4.3. NMR Spectral Analyses

For-proton ^1^H, and Distortionless-Enhancement by-Polarization-Transfer-Q- (DEPT-Q) ^13^C NMR-analyses, a Bruker-Advance-III-400 MHz-with-BBFO-Smart-Probe and a Bruker-400-MHz-EON Nitrogen-Free-Magnet (Bruker-AG, Billerica, MA, USA) was used. For ^1^H and ^13^C measurements, spectra-were-recorded at 400 and 100 MHz, respectively, with tetramethylsilane (TMS) used as an internal standard in chloroform (CDCl_3_) and dimethyl sulfoxide (DMSO-d6). As references, the residual solvent peaks (H = 7.2) and (H = 2.50 ppm and C = 39.5 ppm) were identified. A DEPT-Q experiment was used to determine carbon multiplicities.

### 4.4. Sample Preparation and Extraction

The analyses of the fish samples were carried out in accordance with the AOAC procedure (2006) [62]. The fish sample (1000 g) was thawed, beheaded, de-skinned, and the skin (250 g) washed with water before being ground on an OC-60B/60B grinding-machine- (60–120 mesh, Luohe, China). Extraction was carried out using MeOH (2 L, 2×, three days each). The extract was then chilled, filtered, and dehydrated-by adding-sodium-sulphate anhydrous. Finally, the extract was concentrated by solvent evaporation for 24 h at room temperature, yielding 25 g of crude extract. Following that, 15 g of crude extract were used in the biological study, and 10 g of dried extract was re-suspended in 30 mL of distilled water and defatted with n-Hex. In each step, the organic phase was treated as before, and then evaporated under reduced pressure to yield fractions I (5.0 g), while the aqueous remaining mother liquor was also concentrated to yield fraction (II). All fractions were kept at 4 °C until further phytochemical studies were carried out.

### 4.5. Animal Preparation 

In this case, 25 male Wistar rats (weight 200 ± 20 g) were obtained from the Animal House of the National Research Centre in Egypt. Animals were-placed in cages-under controlled-conditions (12 h of light/dark cycles, temperatures-of 22 ± 1 °C, and humidity-of 40–60%), with ad libitum feeding. The-National Research Centre’s Ethics Committee approved all animal treatments and experimental-procedures with ethical approval no: 5449102021.

### 4.6. MSU-Crystal Synthesis

Uric acid (0.8 g) was dissolved in 155 mL Aquabidest, which also contained 5 mL NaOH (1 M), and the-pH was adjusted-to 7.2 with HCl. Gout-solution was-cooled and stirred at room temperature before being stored overnight at 4 °C to form crystals. After filtering the precipitate from the solution, it was dried at 70 °C for 4 h, ground into a fine powder, sieved through a 200-mesh metal filter, sterilized by heating at 180 °C for 2 h, and stored in sterile conditions. MSU-crystals were suspended-in phosphate-buffered saline (pH 7.2) at a-concentration of 20 mg/mL prior to-administration [63].

### 4.7. Gouty-Arthritis-Animal Model

The development of arthritis was assessed by measuring the size of the joint immediately before the injection with an MK-550 volume meter. Subsequent measurements of the same ankle joint were taken 24 h after the injection and 7 days later. 

After anesthesia with 10% intraperitoneal chloral hydrate (3.5 mL/kg), each rat in the experimental groups (2–5), received 50 μL of MSU solution (20 mg/mL) injected into the left ankle joint cavity. 

**Figure 6:**
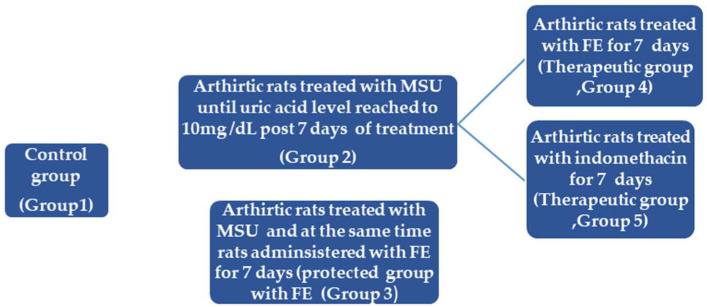
Diagrammatic representation clarifying experimental design. MUS: monosodium urate induced arthritic Wistar albino male rats’ joints. FE: fish’s skin crude extract.

For the experimental protocol, 25 rats were randomly divided into five groups of five rats each: Group 1: Negative control, each animal received 50 μL injection of saline into the left ankle joint cavity. The remain 20 rats were injected with 50 μL of MSU solution (20 mg/mL) for 7 days into the left ankle joint cavity to induce arthritis until the uric acid level reached 10 mg/dL (as determined by a blood sample taken from the retro-orbital plexus) and ankle swelling was measured at the end of 7 days [64]. Then the groups were classified into: Group 2: MSU-induced arthritic rats (positive control), Group 3: MSU + FE (300 mg/kg.b.wt/day; [65], protective group, where rats in this group received FE (300 mg/kg.b.wt/day) combined treatment at the same time of MSU-injection, daily for 7 days). Groups 4: MSU-induced arthritic rats for 7 days, then at the end of MSU injection, rats were treated with FE (300 mg/kg.b.wt/day) for another 7 days (Therapeutic group). Group 5: MSU-induced arthritic rats for 7 days, then post MSU-injection, rats were treated with reference drug indomethacin, 5 mg/kg.b.wt/day [63]) for another 7 days (Therapeutic reference group) (Figure 6). At the end of the experiment, the synovial tissue of the joint was evacuated and partially homogenized and centrifuged, yielding a supernatant that was stored at 20 °C for the examination. 

### 4.8. Biochemical Assays 

TNF-α, IL-1β, NF-𝜅B levels in serum were determined using an ELISA kit and the manufacturer’s instructions. Rat NF kappaB p65 ELISA Kit (ab176648), was used and obtained from Abcam, United states. Rat TNF alpha ELISA kit (ab181421) is a single-wash 90 min sandwich ELISA designed for the quantitative measurement of TNF alpha protein (Abcam, united states), Rat IL-1 beta ELISA Kit (ab255730) is a single-wash 90 min sandwich ELISA designed for the quantitative measurement of IL-1 beta protein in serum, cell culture and plasma. The Lowry Method (1951) [66] was used to determine the protein levels in samples. For histopathological examination of synovial tissue, a portion of it was fixed in 4% paraformaldehyde buffer. In addition, immunohistochemistry was used to quantify inflammatory markers.

Ohkawa et al. (1979) [67] described a method for measuring lipid peroxide (Malondialdehyde; MDA). Ellman (1959) [68] was used to measure the concentration of reduced glutathione (GSH). Glutathione reductase was measured using the method described by Hsiao et al. (2001) [69]. Fridovich (1989) [70] described a method for measuring Superoxide Dismutase (SOD) activity. The Sinha (1972) method was used to determine catalase activity [71]. 

### 4.9. Blood Sampling 

Blood samples were drawn from the retro-orbital plexus and centrifuged at 3000 rpm for 15 min to determine uric acid levels in different groups.

### 4.10. Histopathological Examination

Tissue samples from various experimental groups were collected, fixed in 10% neutral buffered formalin, washed, decalcified with EDTA, dehydrated, cleared, and embedded in paraffin. For histopathological examination, the paraffin-embedded blocks were sectioned at 5-micron thickness and stained with Hematoxylin and Eosin [72]. A light microscope (Olympus BX50, Ina, Japan) was used to examine stained sections).

### 4.11. Histopathological Lesion Scoring

Histopathological alterations were recorded and scored as no changes (0), mild (1), moderate (2), and severe (3) changes. Grading was determined by percentage as follows: 30% change (mild change), 30–50% change (moderate change), and >50% change (severe change) [73].

### 4.12. Immunohistochemistry

The immunohistochemical analysis was performed in accordance to the methods described by Madkour et al., 2021 [74]. Tissue sections were deparaffinized in xylene and rehydrated in various alcohol grades. Antigen retrieval was accomplished by pretreating the sections for 20 min with citrate buffer at pH 6. In a humidified chamber, sections were incubated for two hours with rabbit polyclonal anti-iNOS antibody (ab15323; Abcam, Cambridge, UK). The sections were incubated with goat anti-rabbit IgG H&L (HRP) (ab205718; Abcam, Cambridge, UK), and the chromogen was 3,3′-diaminobenzidine tetrahydro-chloride (DAB, Sigma). The slides were then counterstained with hematoxylin and DPX mounted. PBS was used to replace the primary antibodies in the negative control slides. 

### 4.13. Evaluation of iNOS Immunostaining

In each group, the quantitative immunoreactivity of iNOS was assessed in tissue sections [75], with five tissue sections examined. Immunoreactivity was assessed in 10 microscopical fields per section using a high-power microscope (×400). Color deconvolution image J 1.52 p software (Wayne Rasband, National Institutes of Health, Ina, Japan) was used to calculate the percentage of positively stained cells (%).

### 4.14. Preparation of Fatty Acids Methyl Esters

Methylation was carried out [69]. In a nutshell, 5 mg of fraction I was suspended in 1 mL of n-hexane. Next, in vials, a 2 mL aliquot of methanolic sulfuric acid (1%, *v*/*v*) was added and sealed. For 16 h, the sample was heated in a stopper tube at 50 °C. 2 mL aqueous sodium bicarbonate (2%, *w*/*v*) was added to finish the reaction. The products were then extracted using *n*-hexane (2.5 mL). Finally, samples were concentrated at room temperature for 48 h to remove acids.

### 4.15. GC-MS Analysis of Fatty Acids Methyl Esters

The recovered fatty acid methyl esters were chromatographically analyzed using GC-MS [76]. TRACE^®^ GC Ultra Gas Chromatograph (Thermo Scientific Corp., Berkeley, MO, USA) was used in conjunction with a Thermo MS detector (ISQ^®^ Single Quadrupole Mass Spectrometer, Thermo Fisher Scientific, Berkeley, MO, USA). The system included a TR-5 MS column (30 m × 0.32 mm i.d., 0.25 m film thickness).

The system was set up to analyze 1 L diluted samples (1:10 hexane, *v*/*v*), helium as the carrier gas, and the injector and detector at 210 °C. The flow rate was set to 1.0 mL/min with a split ratio of 1:10. The temperature program was 60 °C for 1 min, then rose at 4.0 °C/min to 240 °C for 1 min. Electron ionization (EI) at 70 eV yielded mass spectra with a spectral range of *m*/*z* 40–450. Finally, the obtained MS data were de-convoluted using AMDIS software (www.amdis.net, accessed on 20 October 2021) and identified by retention indices (relative to n-alkanes C8-C22), mass spectrum matching to authentic standards (when available), and Wiley spectral library collection and NIST library database.

### 4.16. Isolation and Purification of Compounds

Normal vacuum liquid chromatography (VLC) was used to fractionate the *n*-hexane fraction (4 g) using column 6 × 30 cm, 50 g *n*-hexane:EtOAC mixtures were used for gradient elution. The collected fractions (100 mL each) were concentrated and monitored by TLC using the *n*-hexane:EtOAC (8:2) system, and PAA was used to visualize them. Three sub-fractions were created by grouping and concentrating similar fractions (I_1_–I_3_). Subfraction I_1_ (1.50 g) was further fractionated by column chromatography on silica gel 60 (100 × 1 cm, 50 g), which was eluted as before to yield compounds **1** (20 mg) and **2** (10 mg), whereas subfractions I_2_ and I_3_ (1.00 g, each) yielded compounds **3** (50 mg) and **4** (30 mg). 

### 4.17. Amino Acid Analysis 

Fraction II (4 g) was also further using for amino acid analysis as follow:

#### 4.17.1. Device Specification

Sykam Amino Acid Analyzer (Sykam GmbH, Eresing, Germany) outfitted with Solvent Delivery System S 2100 (Quaternary pump with flow range 0.01 to 10.00 mL/min and maximum pressure up to 400 bar), Autosampler S 5200, Amino Acid Reaction Module S4300 (with built-in dual filter photometer between 440 and 570 nm with constant signal output and signal summary option) and Refrigerated Reagent Organizer S 4130.

#### 4.17.2. Standard Preparation

The stock solution contains 18 amino acids (aspartic acid, threonine, serine, glutamic acid, proline, glycine, alanine, cystine, valine, methionine, isoleucine, leucine, tyrosine, phenylalanine, histadine, lysine, ammonia, arginine) with concentrations of 2.5 mol/mL, except cystine 1.25 mol/mL, then dilute 60 µL in 1.5 mL vial with sample dilution buffer then filtered using 0.22 µm syringe filter then 100 µL was injected.

#### 4.17.3. Sample Preparation

300 mg of the sample was combined with 5 mL of hexane. For 24 h, the mixture was allowed to macerate. The mixture was then filtered through Whatman no. 1 filter paper, and the residue was transferred to a test tube and incubated in an oven with 10 mL 6N HCl for 24 h at 110 °C. Following incubation, the sample was filtered on Whatman no. 1 filter paper, evaporated on a rotary evaporator, and completely dissolved in 100 mL dilution buffer before diluting 1 mL in 3 mL vial, filtered using 0.22 m syringe filter, and 100 µL was injected.

#### 4.17.4. Instrument Parameters

LCA K06/Na column, buffer a, buffer b, and the regeneration solution are all part of the mobile phase. Gradient elution mode 0.45 mL/min flow rate temperature: 57–74 °C gradient, wavelength: 440 and 570 nm, preparation of buffers and solutions (Table 9):

### 4.18. Molecular Modeling Study

#### 4.18.1. Ligand Preparation

Using the AutodockTools v.4.2 set, all torsions of the isolated compound structures were assigned and their Gasteiger charges were provided for all investigated atoms in structures [77]. Structures that had more than 32 torsions were eliminated.

#### 4.18.2. Receptor Preparation

Docking screening was performed on human COX-1 and COX-2 structures with co-crystallized ligands CEL and arachidonic acid (PDB codes: 3kk6 and 3hs5, respectively). PDBfixer [78] was used to repair missing residues and atoms, as well as to remove co-crystalized H2O and hetero-atoms from the downloaded structure. Following that, AutodockTools v.4.2 was used to provide polar hydrogen and Gasteiger charge to all receptors [77].

#### 4.18.3. Structural Docking

For the docking step, the PyRx platform’s Auto-Dock Vina software was used [79,80]. The docking search binding sites were estimated based on the enzyme’s co-formed ligands (i.e., CEL and arachidonic acid for COX-1 and COX-2, respectively). For the active sites of COX-1 and COX-2, the grid box co-ordinates were *x* = −31.94; *y* = 42.74; *z* = −4.39, and *x* = 20.95; *y* = 15.73; *z* = 66.82, respectively. The grid box size was set to 15 while the exhaustiveness was set to 24. Pymol software was used to analyze and visualize docking poses [80].

#### 4.18.4. Molecular Dynamics Simulation

MDS experiments were carried out using the Desmond v. 2.2 software [81,82,83,84]. The OPLS-2005 force field is used in this software. Protein systems were created using the System Builder option, which checked the protein structure for any missing hydrogens, set the protonation states of the amino acid residues (pH = 7.4), and removed the co-crystalized water molecules. Following that, the entire structure was immersed in an orthorhombic box of TIP3P water containing 0.15 M Na^+^ and Cl^−^ ions in a solvent buffer of 20. Following that, the ready systems were energy minimized and equilibrated for 10 ns. The top-scoring poses for protein-ligand complexes were used as starting points for simulation. During the system building step, the Desmond software automatically parameterizes inputted ligands based on the OPLS force field. The absolute binding free energy (Gbinding) was determined using NAMD simulations [85], and the protein structures were built and optimized using the VMD software’s QwikMD toolkit. The compounds’ parameters and topologies were calculated using the VMD plugin Force Field Toolkit (ffTK). Following that, the generated parameters and topology files were loaded into VMD to easily read the protein-ligand complexes and then run the simulation steps.

#### 4.18.5. Absolute Binding Free Energy Calculations

The free energy perturbation (FEP) method was used to calculate binding free energy (G). Kim and colleagues [86] recently published a detailed description of this method. The value of each G is estimated using NAMD software from a separate simulation. The online website Charmm-GUI (https://charmm-gui.org/?doc=input/afes.abinding, accessed on 18 May 2021) can be used to prepare all input files required for NAMD simulation). Following that, we can use these files in NAMD to generate the required simulations using the FEP calculation function. In the presence of the TIP3P water model, the equilibration (5 ns long) was achieved in the NPT ensemble at 300 K and 1 atm (1.01325 bar) with Langevin piston pressure (for “Complex” and “Ligand”). Next, for each compound, 10 ns FEP simulations were run, and the final 5 ns of free energy values were measured for the final free energy values [86].

### 4.19. Statistical Analyses

Statistical analysis is performed using the SPSS computer program (One Way Analysis of Variance, ANOVA) in conjunction with the co-state computer program, with different letters being significant at *p* ≤ 0.05% Change is calculated compared to control group as: (mean of treated − mean of negative/mean of negative control) × 100%. Reduction: (mean of positive control—mean of treated group/mean of positive control) × 100%.

## 5. Conclusions

Nutrition has a significant impact on a person’s health. Omega-3 fatty acids and protein-rich FE have been shown to reduce inflammation. Not only because of the absence of harmful side effects and the positive health benefits of FE, but also because of the exceptional therapeutic impact demonstrated in this study in arthritis-induced rats. *M. electricus* FE effects could be attributed to inhibitory and modulatory actions on the production and release of nitric oxide and cytokines, both of which are involved in disease pathogenesis. According to molecular modelling and dynamics simulation, one of the major components of the crude extract (compound **4**) has the potential to target and inhibit COX isoforms with a higher affinity for COX-2. For all of this, we aim that the FE will provide an impressive and encouraging therapy option for arthritis, contributing to the reduction and progression of this chronic-inflammatory disorder.

## Figures and Tables

**Table 1:** Effects of *Malapterurus electricus* skin extract on uric acid levels, and ankle swelling reduction in serum of MSU Crystal-Gouty Arthritis Rats.

Groups	Uric Acid (mg/dL)	Ankle Swelling (mm) Post 24 h	Ankle Swelling (mm) Post 7 Says
Control Group	3.00 ± 0.23 ^d^	0.50 ± 0.03 ^a^	0.50 ± 0.02 ^a^
Msu Group % Change	10.90 ± 0.30 ^a^ 263.33	3.55 ± 0.12 ^b^ 610.00	6.55 ± 0.56 ^b^ 1210
Msu + Fe % Reduction	5.00 ± 2.20 ^c^ 54.13	1.32 ± 0.44 ^c^ 62.82	1.90 ± 0.9 ^c^ 70.99
Msu treated with Fe % Reduction	4.00 ± 0.90 ^b^ 63.30	1.95 ± 0.66 ^d^ 45.10	1.89 ± 0.11 ^c^ 71.15
Msu treated Standard Drug % Reduction	3.50 ± 0.60 ^c^ 67.89	1.00 ± 0.43 ^e^ 71.83	1.80 ± 0.20 ^c^ 72.52

Data are mean ±SD of five rats in each group. Statistical analysis is brought out utilizing SPSS computer program (One-way Analysis-of-Variance, ANOVA; IBM SPSS (version 8) (SPSS Inc., Chicago, IL, USA) connected with co-state computer program, where different letters at the same column are significant at *p* ≤ 0.05. FE: fish’s skin crude extract. % Change is calculated compared to control group as: (mean of treated − mean of negative/mean of negative control) × 100. % Reduction: (mean of positive control − mean of treated group/mean of positive control) × 100.

**Table 2:** Effects of *Malapterurus electricus* skin extract *on* inflammatory markers TNF-α, IL-1β, and NF-𝜅B in Synovial Tissues homogenate.

	TNF-α (pg/mL)	IL-1β (pg/mL)	NF-𝜅B (pg/mL)
Control Group	89.90 ± 9.00 ^a^	17.55 ± 1.40 ^a^	0.033 ± 0.003 ^a^
Msu Group % Change	700.00 ± 30.00 ^b^ 678.64	190.00 ± 19.00 ^b^ 982.62	0.10 ± 0.03 ^b^ 203.03
Msu + FE % Reduction	340.00 ± 16.90 ^c^ 51.43	89.00 ± 11.00 ^c^ 53.16	0.050 ± 0.02 ^c^ 50.00
Msu treated Fe % Reduction	200.00 ± 10.00 ^d^ 71.43	50.00 ± 3.00 ^d^ 73.68	0.040 ± 0.02 ^d^ 60.00
Msu treated Standard Drug % Reduction	190.00 ± 8.10 ^d^ 72.86	45.00 ± 2.90 ^d^ 76.32	0.037 ± 0.03 ^d^ 63.00

Data are mean ± SD of five rats in each group. Statistical analysis is brought out employing SPSS computer program (One-way Analysis-of-Variance, ANOVA; IBM SPSS (version 8) (SPSS Inc., Chicago, IL, USA) connected with co-state computer program, where different letters at the same column are significant at *p* ≤ 0.05. FE: fish’s skin crude extract, TNF-α: Tumor-Necrosis-Factor Alpha, IL-1β: Interleukin-1-beta, NF-κB: Nuclear-factor-kappa-B. % Change is calculated compared to control group as: (mean of treated − mean of negative/mean of negative control) × 100. % Reduction: (mean of positive control − mean of treated group/mean of positive control) × 100.

**Table 3:** Effects of *Malapterurus electricus* skin extract on oxidative stress and antioxidant markers in Synovial Tissues homogenate.

Groups	GSH Reductase (mmol/min/g Tissue)	Catalase (µmol/min/g Tissue)	GSH (mg/g Tissue)	MDA (µmol/g Tissue)	SOD (µmol/g Tissue)
Control Group	0.08 + 0.01 ^a^	0.09 + 0.02 ^a^	90.00 + 6.00 ^a^	4.10 + 0.18 ^a^	255.00 + 22.0 ^a^
MSU Group % Change	0.04 + 0.02 ^b^ 50.00	0.04 + 0.04 ^b^ 55.56	33.34 + 2.11 ^b^ 62.95	11.20 + 1.00 ^b^ 173.17	105.0 + 9.10 ^b^ 58.82
MSU + FE % Reduction	0.055 + 0.02 ^c^ 37.50	0.070 + 0.03 ^c^ 75.00	57.00 + 4.10 ^d^ 70.96	7.80 + 0.90 ^c^ 30.36	168.0 + 9.15 ^c^ 60.00
MSU Treated FE % Reduction	0.079 + 0.03 ^a^ 97.50	0.080 + 0.05 ^a^ 100.00	82.00 + 4.66 ^a^ 145.95	5.14 + 0.59 ^a^ 54.11	190.00 + 10.0 ^c^ 80.95
MSU Treated Standard Drug % Reduction	0.064+ ±0.04 ^d^ 60.00	0.080 + 0.02 ^a^ 100.00	80.00 + 3.50 ^a^ 139.95	5.48 + 0.66 ^a^ 51.10	188.10 + 10.00 ^c^ 79.14

Data are mean ± SD of five rats in each group. Statistical analysis is brought out utilizing SPSS computer program (One-way Analysis-of-Variance, ANOVA; IBM SPSS (version 8) (SPSS Inc., Chicago, IL, USA) connected with co-state computer program, where different letters at the same column are significant at *p* ≤ 0.05. FE: fish’s skin crude extract, GSH: Glutathione, MDA: malondialdehyde, SOD: Superoxide dismutase. % Change is calculated compared to control group as: (mean of treated—mean of negative/mean of negative control) × 100. % Reduction: (mean of positive control—mean of treated group/mean of positive control) × 100.

**Table 4:** Scoring of histopathological alterations of all treated groups.

Lesions	G1	G2	G3	G4	G5
Irregularity and deformities of articular surface	0	3	0	1	1
Necrosis of chondrocytes	0	3	1	1	1
Synovial membrane infiltrated with inflammatory cells	0	3	1	1	1
Widening of trabecular space	0	3	0	1	1

The score process was composed as: score 0 = lack of the lesion in all rats of the group (*n* = 5), score 1 = (<30%), score 2 = (<30%–50%), score 3 = (>50%). G1: control rats, G2: Arthritic rats, G3: Arthritic rats co-administered with extract, G4 and G5: Arthritic rats treated with fish extract and standard drug respectively.

**Table 5:** % Expression of iNOS of different experimental groups.

Groups	G1	G2	G3	G4	G5
Affected Area					
Articular surface	0	41.10 ± 1.50 ^b^	18.00 ± 0.88 ^a^	28.00 ± 3.00 ^c^	31.00 ± 1.80 ^c^
% Reduction to arthritic rats	-	-	56.20	31.87	24.57
Synovial membrane	0	60.15 ± 3.00 ^b^	19.00 ± 1.00 ^a^	31.00 ± 1.00 ^c^	34.00 ± 2.00 ^c^
% Reduction to arthritic rats	-	-	68.41	48.46	43.47

Each value was expressed as mean ± SEM. Different letters in the same column are significantly different (*p* ≤ 0.05). G1: control rats, G2: Arthritic rats, G3: Arthritic rats co-administered with extract, G4 and G5: Arthritic rats treated with fish extract and standard drug respectively. % Reduction is calculated according to arthritic rats using the equation: (mean of disease rats − mean of treated rats/Mean of disease rats) × 100.

**Table 6:** *Malapterurus electricus* fish oil composition using GC/MS analysis.

No.	Identified Compound	C:D	Type	Area %	RT	RI
	Palmitoleic acid	C16:1 (9)	MUFA	1.70	27.81	919
1	Palmitic acid	C16:0	SFA	34.66 *	28.68	931
2	Vaccenic acid	C18:1 (11)	MUFA	24.52 *	32.64	931
	9-Octadecenoic acid	C18:1 (9)	MUFA	11.66	32.75	946
3	Stearic acid	C18:0	SFA	14.63	33.24	935
	Arachidonic acid	C20:4 (5,8,11,14)	PUFA	3.41	35.83	913
	5,8,11,14,17-Eicosapentaenoic acid	C20:5 (5,8,11,14,17)	PUFA	1.33	35.94	905
4	4,7,10,13,16,19-Docosahexaenoic acid	C22:6 (4,7,10,13,16,19)	PUFA	6.00	39.78	938
SFA				49.29%		
MUFA				37.88%		
PUFA				10.74%		
Total				97.91%		

RI: retention-index relative to *n*-alkanes, RT: retention-time for fatty-acid in ester form (min), C:D: carbon-number to double-bond-number covering their position, *: major compound, SFA: saturated-fatty-acid, MUFA: mono-unsaturated-fatty-acid, PUFA: poly-unsaturated-fatty-acid, %: percentage.

**Table 7:** Amino acid contents of *Malapterurus electricus* extract.

Identified Compound	RT	Amount (mg/100 mg)
Aspartic Acid	7.699	0.142
Threonine	9.816	0.144
Serine	10.549	0.103
Glutamic	11.915	0.138
Proline	13.933	0.289
Glycine	17.824	0.813
Alanine	19.056	1.645 *
Cystine	21.264	0.034
Valine	21.915	0.206
Methionine	23.803	0.019
Isoleucine	25.915	0.108
Leucine	27.171	0.247
Tyrosine	30.315	0.014
Phenylalanine	31.389	0.124
Histidine	35.128	0.142
Lysine	39.381	0.242
Arginine	43.056	0.173
Total AA		4.584

RT: retention time for amino acid (min), AA: amino acid, *: major compound.

**Table 8:** Docking scores and Δ*G*_binding_ of the isolated fatty acids inside COX-1′ and COX-2′s binding sites.

Fatty Acid	Docking Score		Δ*G*_binding_	
	COX-1	COX-2	COX-1	COX-2
1	−7.41	−7.56	−3.23	−3.57
2	−7.51	−7.34	−5.38	−6.34
3	−7.39	−7.44	−3.86	−3.92
4	−8.46	−8.75	−8.23	−12.45
CEL *	−12.56	−9.45	−10.43	−8.43
Arachidonic acid **	−11.54	−10.53	−9.42	−9.54

* The reported co-crystalized ligand of COX-1. ** The reported co-crystalized ligand of COX-2.

**Table 9:** Preparation of buffers and solutions.

	Buffer A	Buffer B	Column Regeneration Solution	Sample Dilution Buffer
pH Value	3.45	10.85		2.20
Normality	0.12	0.20	0.50	0.12
Tri-sodium citrate dihydrate	11.8 g	19.6 g		11.8 g
NaOH		3.1 g	20.0 g	
Citric acid	6.0 g			6.0 g
Boric acid		5.0 g		
Methanol	65 mL			
Thiodiglycol				14 mL
Hydrochloric acid 32%	6.5 mL			12 mL
EDTA			0.2 g	
Phenol	0.5 g			2.0 g
Final volume	1 L	1 L	1 L	1 L

## Supplementary Materials

The accompanying supporting data can be loaded at: www.mdpi.com/article/10.3390/md20100639/s1, Figure S1: GC/MS spectrum fish oil; Figure S2: 1H-NMR spectrum of compound **1** in DMSO-d6 at 400 MHz; Figure S3: DEPT-Q-NMR spectrum of compound **1** in DMSO-d6 at 100 MHz; Figure S4: 1H NMR spectrum of compound **2** in DMSO-d6 at 400 MHz; Figure S5: DEPT-Q-NMR spectrum of compound **2** in DMSO-d6 at 100 MHz; Figure S6: 1H NMR spectrum of compound **3** in CDCl3 at 400 MHz; Figure S7: DEPT-Q NMR spectrum of compound **3** in CDCl3 at 100 MHz; Figure S8: 1H NMR spectrum of compound **4** in DMSO-d6 at 400 MHz; Figure S9: DEPT-Q NMR spectrum of compound **4** in DMSO-d6 at 100 MHz; Figure S10: Amino acid sample analysis.
