# Supplementary material for: Anti-Inflammatory and Antioxidant Properties of Malapterurus electricus Skin Fish Methanolic Extract in Arthritic Rats: Therapeutic and Protective Effects
Source: Mar Drugs. 2022 Oct 14;20(10):639. doi: 10.3390/md20100639 (PMC9604635; doi:10.3390/md20100639)
Supplement: Supplementary file 1 [file marinedrugs-20-00639-s001.zip › marinedrugs-1915947-supplementary.pdf]

SUPPLEMENTARY DATA

Anti-inflammatory and Antioxidant Properties of *Malapterurus electricus* Skin Fish Methanolic Extract in Arthritic Rats: Therapeutic and protective effects

Abeer H. Elmaidomy[1*], Esraa M. Mohamed[2], Hanan F. Aly[3], Eman A. Younis[3], Shams Gamal Eldin Shams[3], Faisal H. Altemani[4], Mubarak A. Alzubaidi[5], Mohammed Almaghrabi[6], Adnan Al Harbi[7], Faisal Alsenani[8], Ahmed M. Sayed[9], Usama Ramadan Abdelmohsen[10,11*]

[1]Department of Pharmacognosy, Faculty of Pharmacy, Beni-Suef University, Beni-Suef 62511, Egypt
[2]Department of Pharmacognosy, Faculty of Pharmacy, MUST, Giza, 12566, Egypt
[3]Department of Therapeutic Chemistry, Pharmaceutical and Drug Industries Research Institute, National Research Centre, P.O. 12622, El Bouhouth St., Dokki, Giza, Egypt
[4]Department of Medical Laboratory Technology, Faculty of Applied Medical Sciences, University of Tabuk, Tabuk 71491, Saudi Arabia
[5]Department of Biological Sciences, Faculty of Science, King Abdulaziz University, Jeddah 21589, Saudi Arabia
[6]Pharmacognosy and pharmaceutical chemistry department, Faculty of Pharmacy, Taibah University 42353, Al Madinah Al Munawarah, Saudi Arabia
[7]Clinical Pharmacy Department, College of Pharmacy, Umm Al-Qura University, Makkah 21955, Saudi Arabia; assharbi@uqu.edu.sa.
[8]Department of Pharmacognosy, Faculty of Pharmacy, Umm Al-Qura University, Makkah 21955, Saudi Arabia; fssenani@uqu.edu.sa
[9]Department of Pharmacognosy, Faculty of Pharmacy, Nahda University, 62513 Beni-Suef, Egypt
[10]Department of Pharmacognosy, Faculty of Pharmacy, Minia University, Minia 61519, Egypt
[11]Department of Pharmacognosy, Faculty of Pharmacy, Deraya University, 7 Universities Zone, New Minia 61111, Egypt

Corresponding authors: Department of Pharmacognosy, Faculty of Pharmacy, Minia University, Minia 61519, Egypt, usama.ramadan@mu.edu.eg, Department of Pharmacognosy, Faculty of Pharmacy, Beni-Suef University, Beni-Suef 62511, Egypt, Abeer011150@pharm.bsu.edu.eg

Abstract
The protective and therapeutic anti-inflammatory and antioxidant potency of *Malapterurus electricus* (F. Malapteruridae) skin fish methanolic extract (FE) (300mg/kg.b.wt/day for 7days, orally) was tested in monosodium urate(MSU)-induced arthritic Wistar albino male rats' joints. Serum uric acid, TNF-α, IL-1β, NF-κB, MDA, GSH, catalase, SOD, and glutathione reductase levels were all measured. According to the findings, FE significantly reduced uric acid levels and ankle swelling in both protective and therapeutic groups. Furthermore, it has anti-inflammatory effects by downregulating inflammatory cytokines, primarily through decreased oxidative stress and increased antioxidant status. All the aforementioned lesions were significantly improved in protected and treated rats with FE, according to histopathological findings. iNOS immunostaining revealed that protected and treated arthritic rats with FE had weak positive immune-reactive cells. Phytochemical analysis revealed that FE was high in fatty and amino acids. The most abundant compounds were vaccenic (24.52%), 9-octadecenoic (11.66%), palmitic (34.66%), stearic acids (14.63%), glycine (0.813mg/100mg), and alanine (1.645mg/100mg). Extensive molecular modelling and dynamics simulation experiments revealed that compound 4 has the potential to target and inhibit COX isoforms with a higher affinity for COX-2. As a result, we contend that FE could be a promising protective and therapeutic option for arthritis, aiding in the prevention and progression of this chronic inflammatory disease.
Keywords: *Malapterurus electricus;* anti-inflammatory; COX-2; molecular dynamics simulation.

List of Contents

Figure S1. GC/MS spectrum *Malapterurus electricus* skin oil

Figure S2. 1H NMR spectrum of compound **1** measured in DMSO-d_6 at 400 MHz

Figure S3. DEPT-Q NMR spectrum of compound **1** measured in DMSO-d_6 at 100 MHz

Figure S4. 1H NMR spectrum of compound **2** measured in DMSO-d_6 at 400 MHz

Figure S5. DEPT-Q NMR spectrum of compound **2** measured in DMSO-d_6 at 100 MHz

Figure S6. 1H NMR spectrum of compound **3** measured in $CDCl_3$ at 400 MHz

Figure S7. DEPT-Q NMR spectrum of compound **3** measured in $CDCl_3$ at 100 MHz

Figure S8. 1H NMR spectrum of compound **4** measured in DMSO-d_6 at 400 MHz

Figure S9. DEPT-Q NMR spectrum of compound **4** measured in DMSO-d_6 at 100 MHz

Figure S10. Amino acid sample analysis.

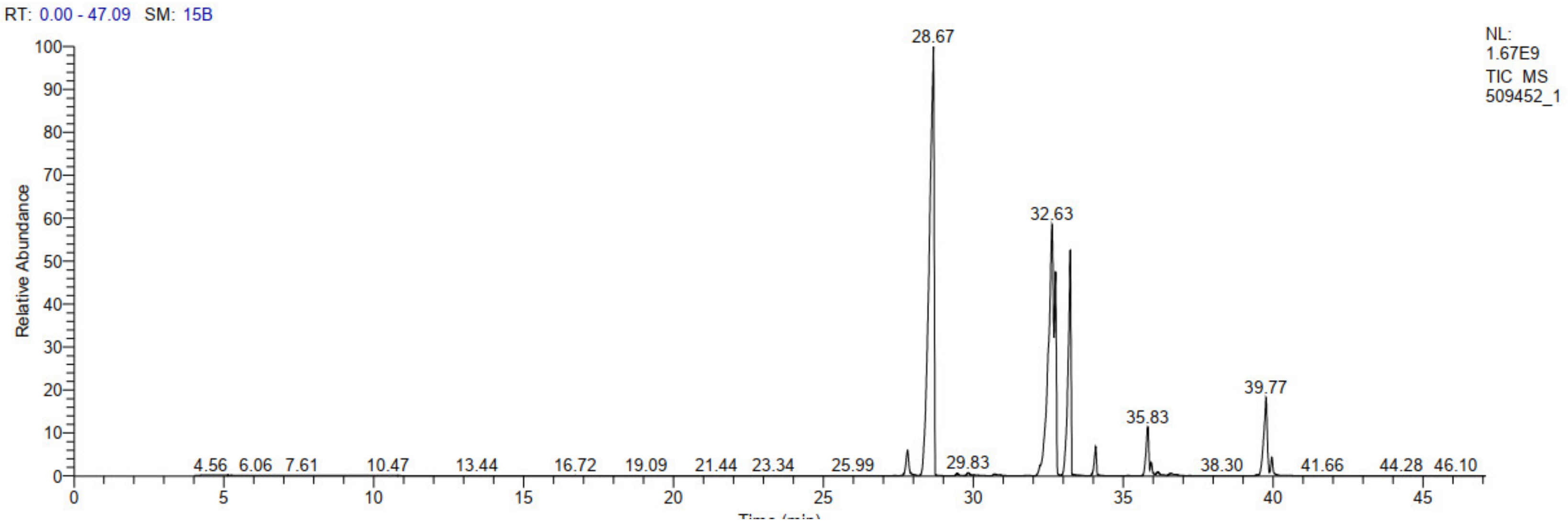

Figure S1. GC/MS spectrum *Malapterurus electricus* skin oil

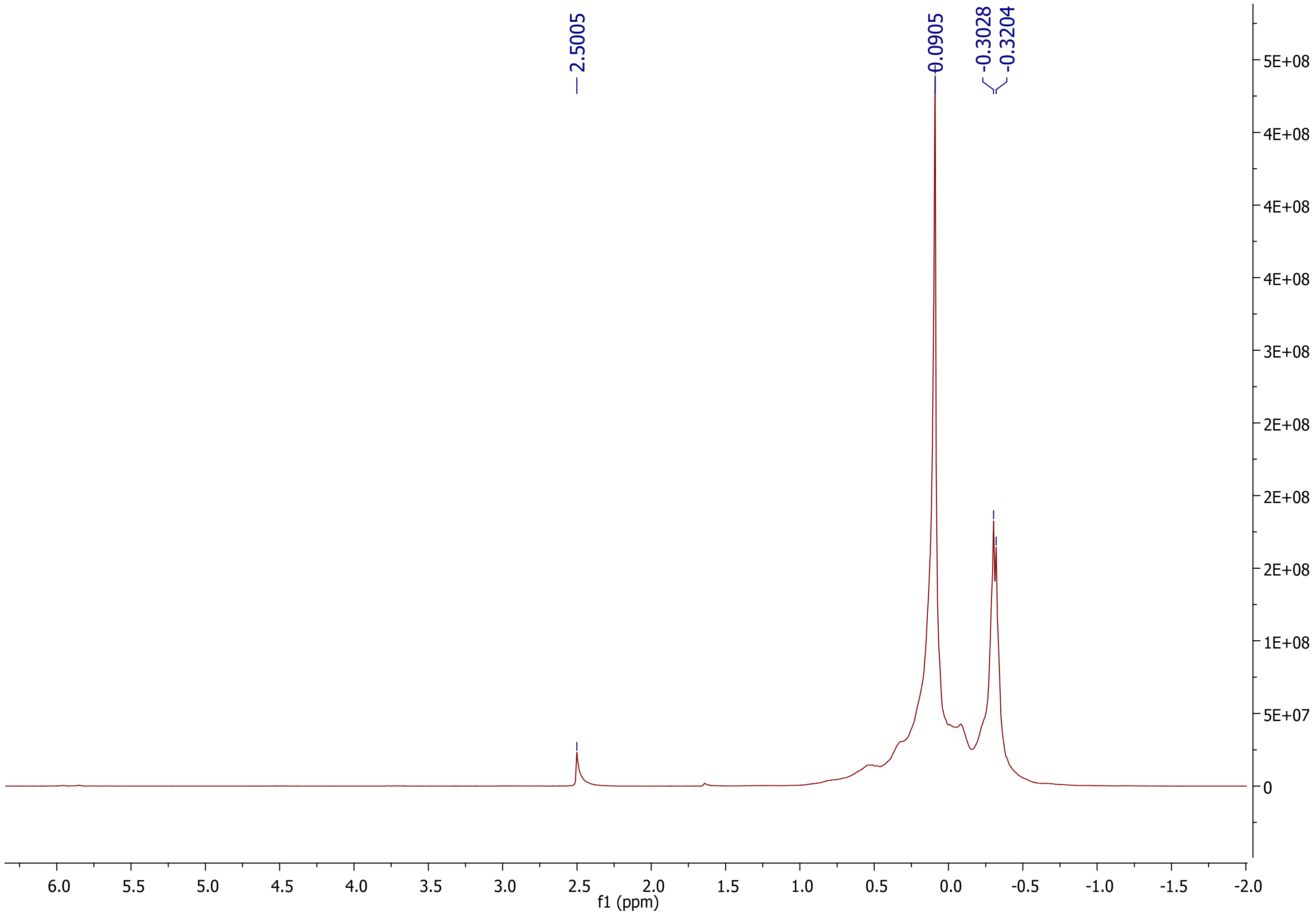

Figure S2. 1H NMR spectrum of compound **1** measured in DMSO-d_6 at 400 MHz

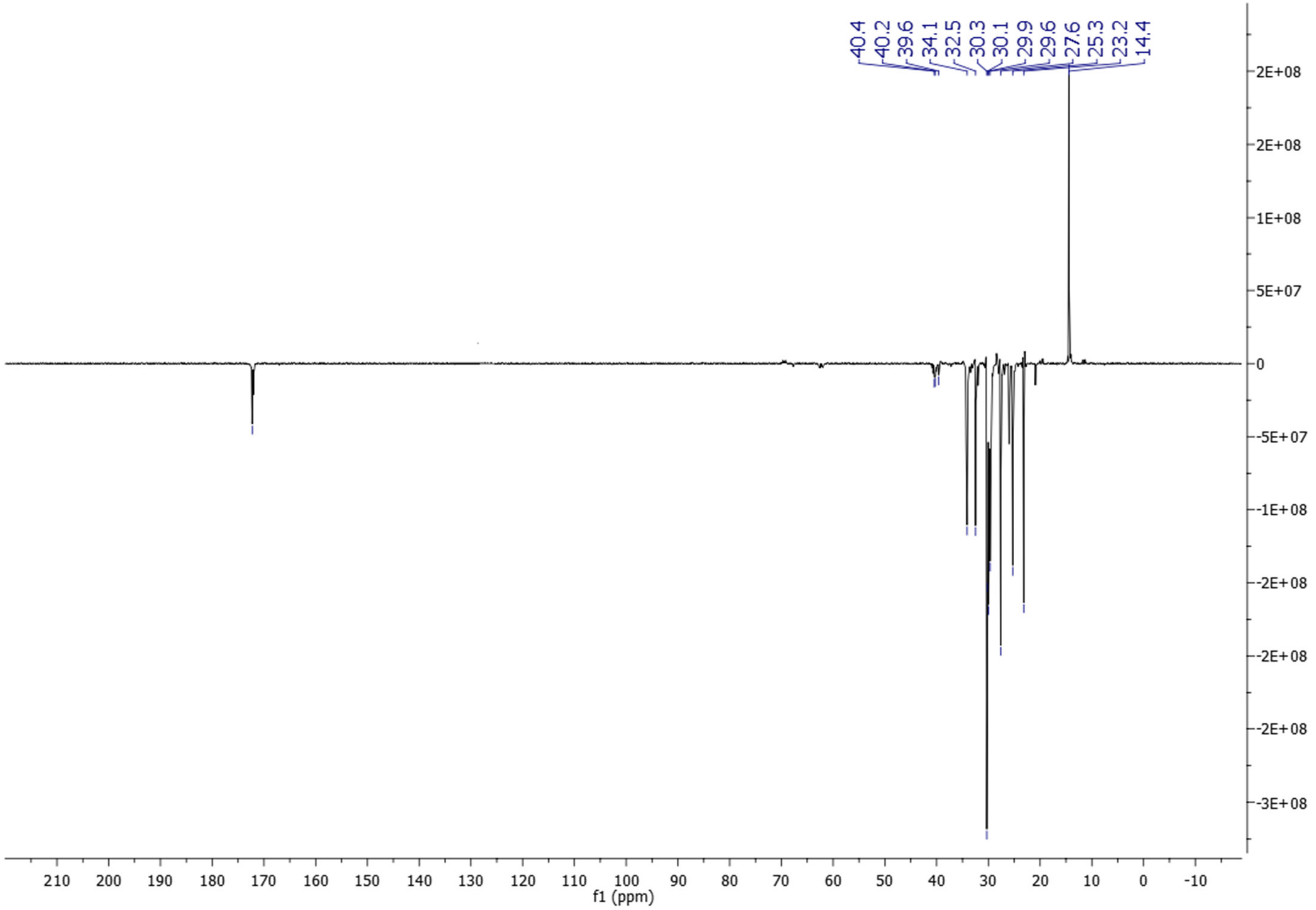

Figure S3. DEPT-Q NMR spectrum of compound **1** measured in DMSO-d_6 at 100 MHz

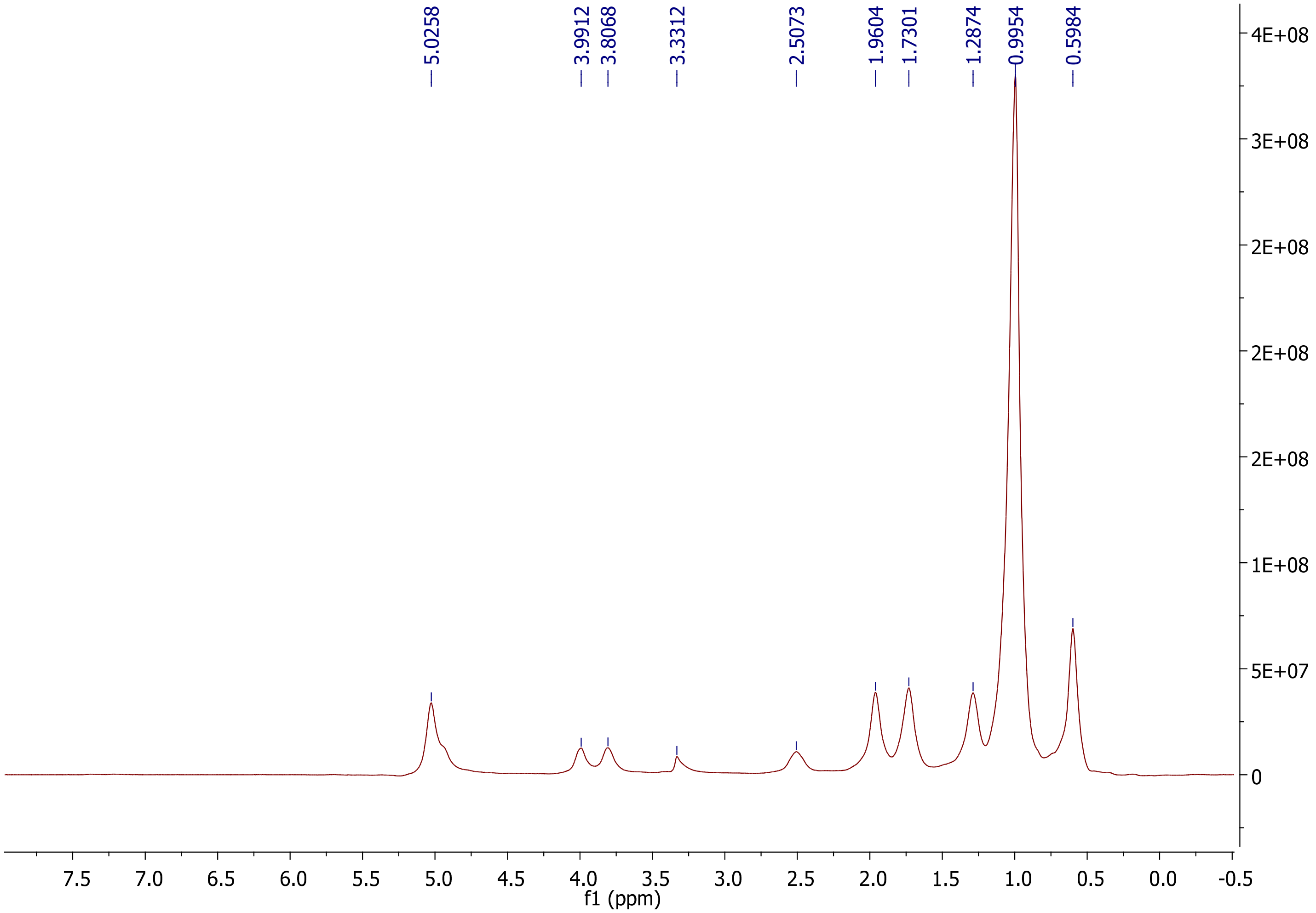

Figure S4. 1H NMR spectrum of compound **2** measured in DMSO-d_6 at 400 MHz

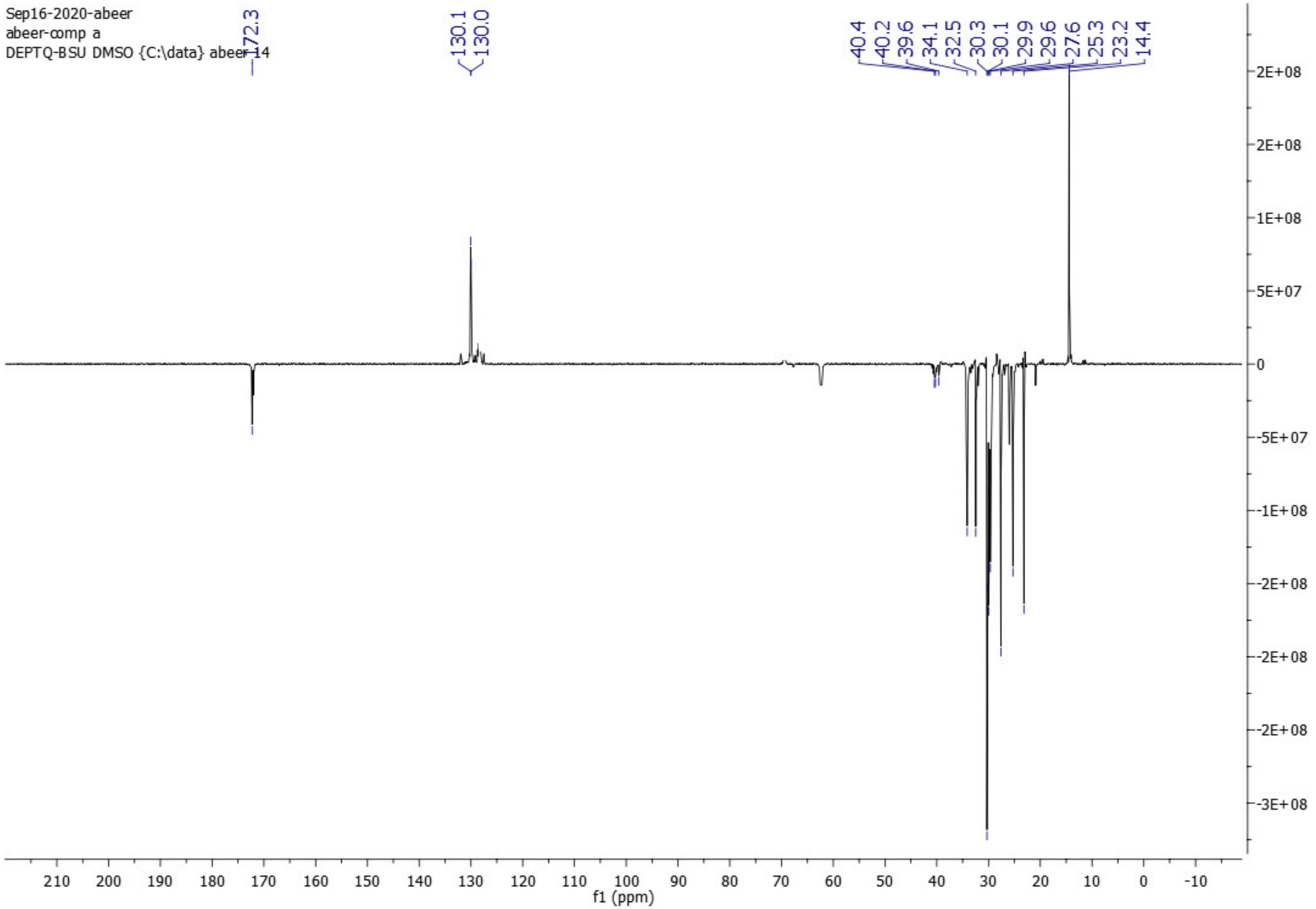

Figure S5. DEPT-Q NMR spectrum of compound **2** measured in DMSO-d_6 at 100 MHz

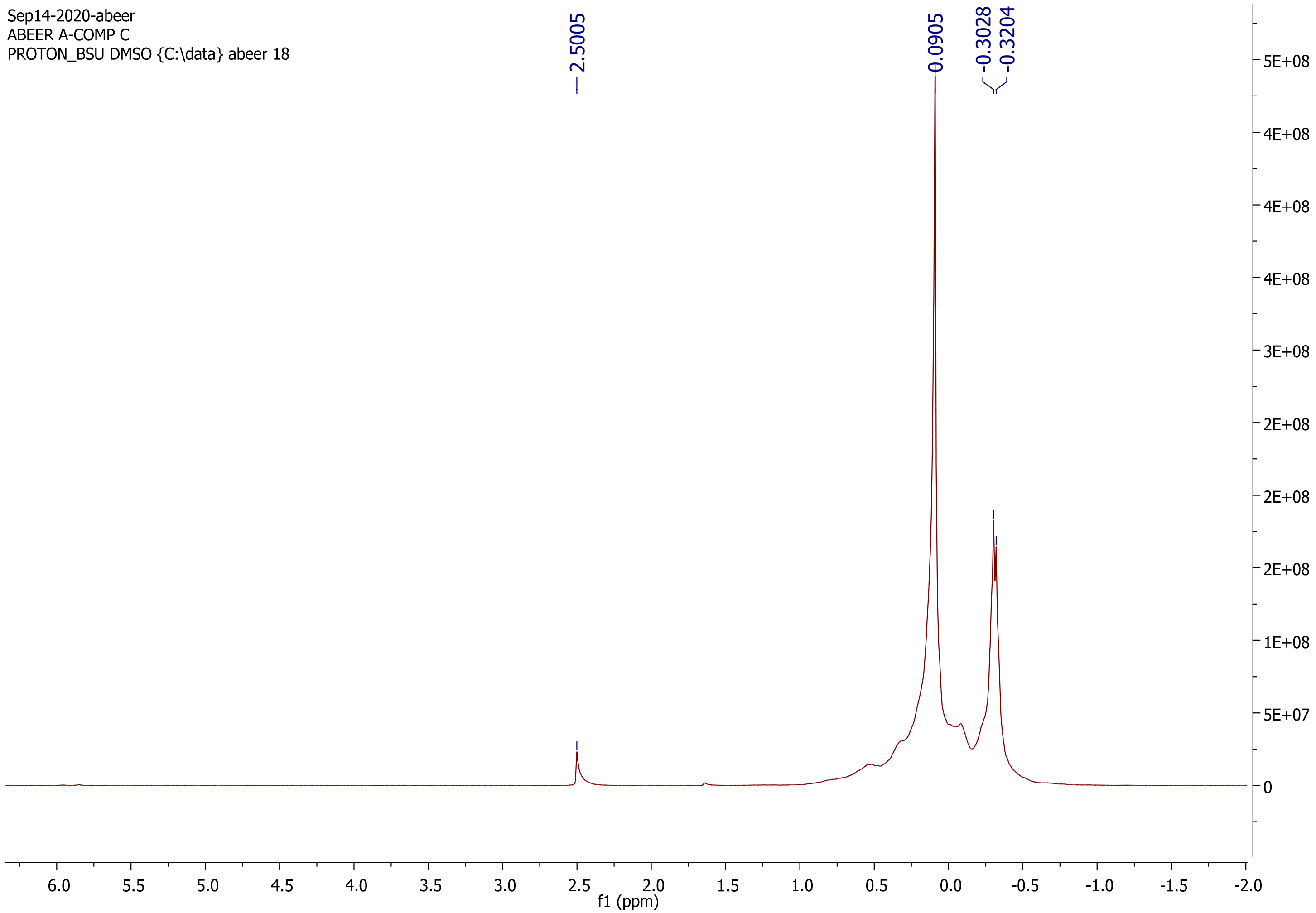

Figure S6. 1H NMR spectrum of compound **3** measured in DMSO-d_6 at 400 MHz

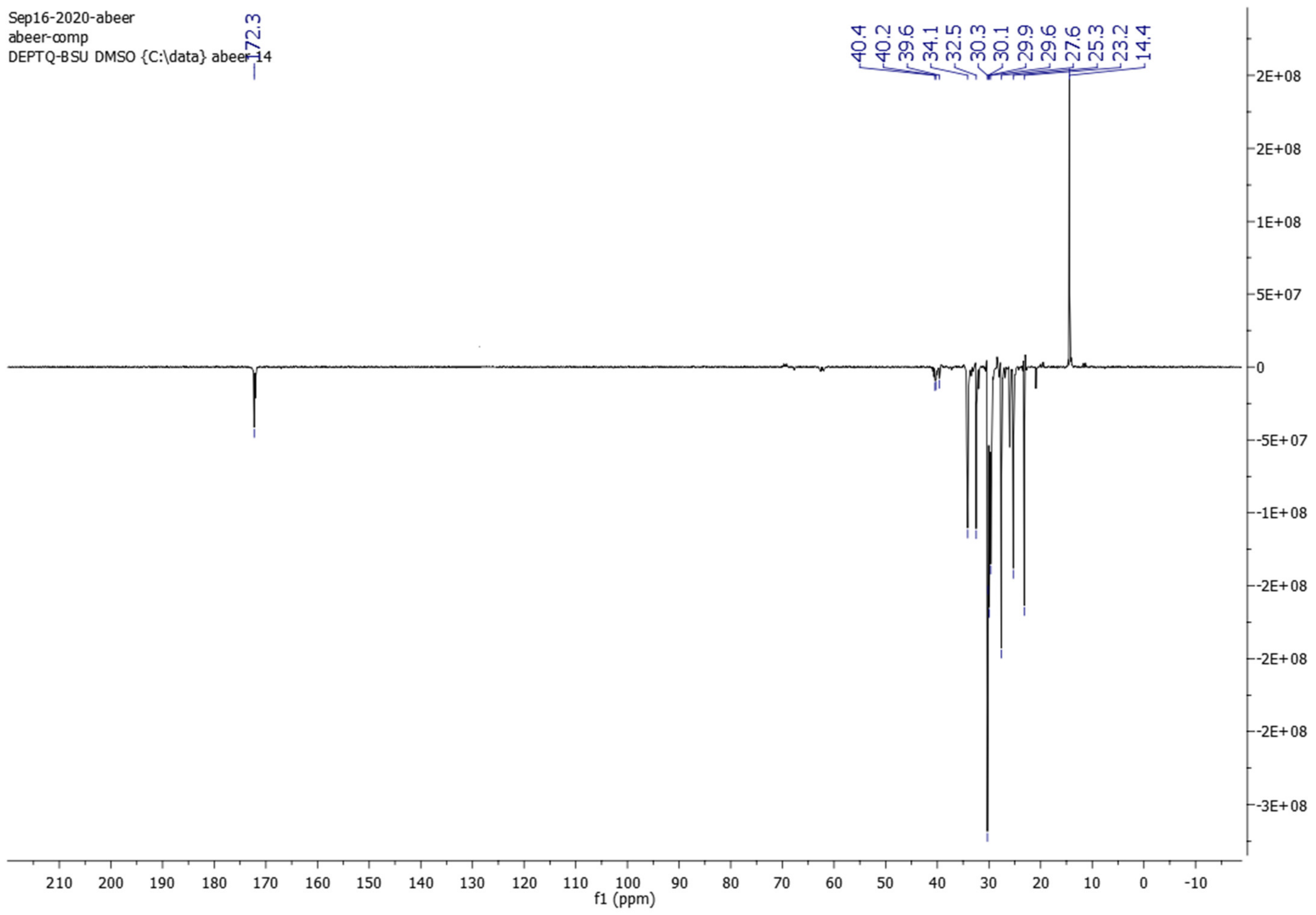

Figure S7. DEPT-Q NMR spectrum of compound **3** measured in DMSO-d_6 at 100 MHz

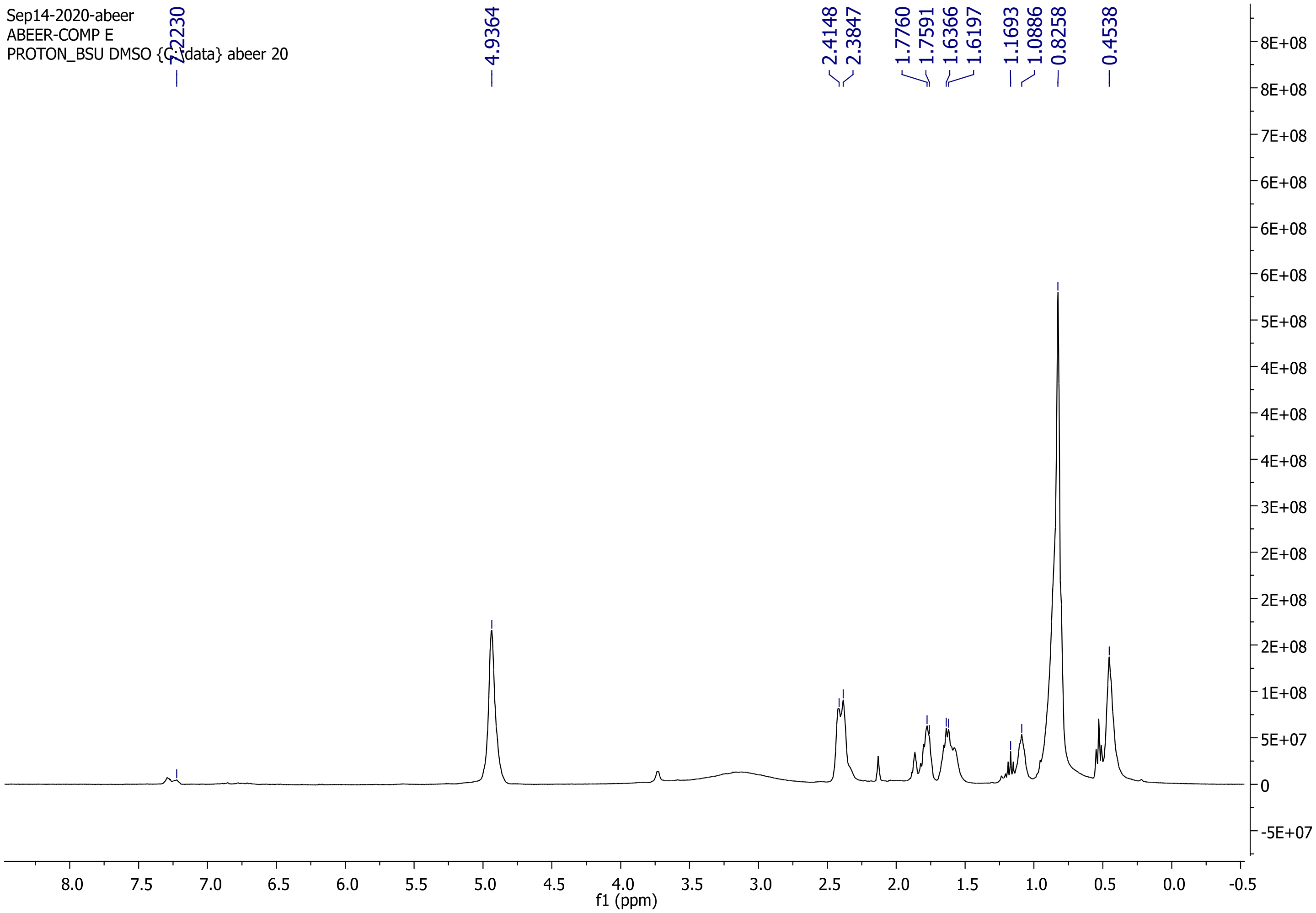

Figure S8. 1H NMR spectrum of compound **4** measured in DMSO-d_6 at 400 MHz

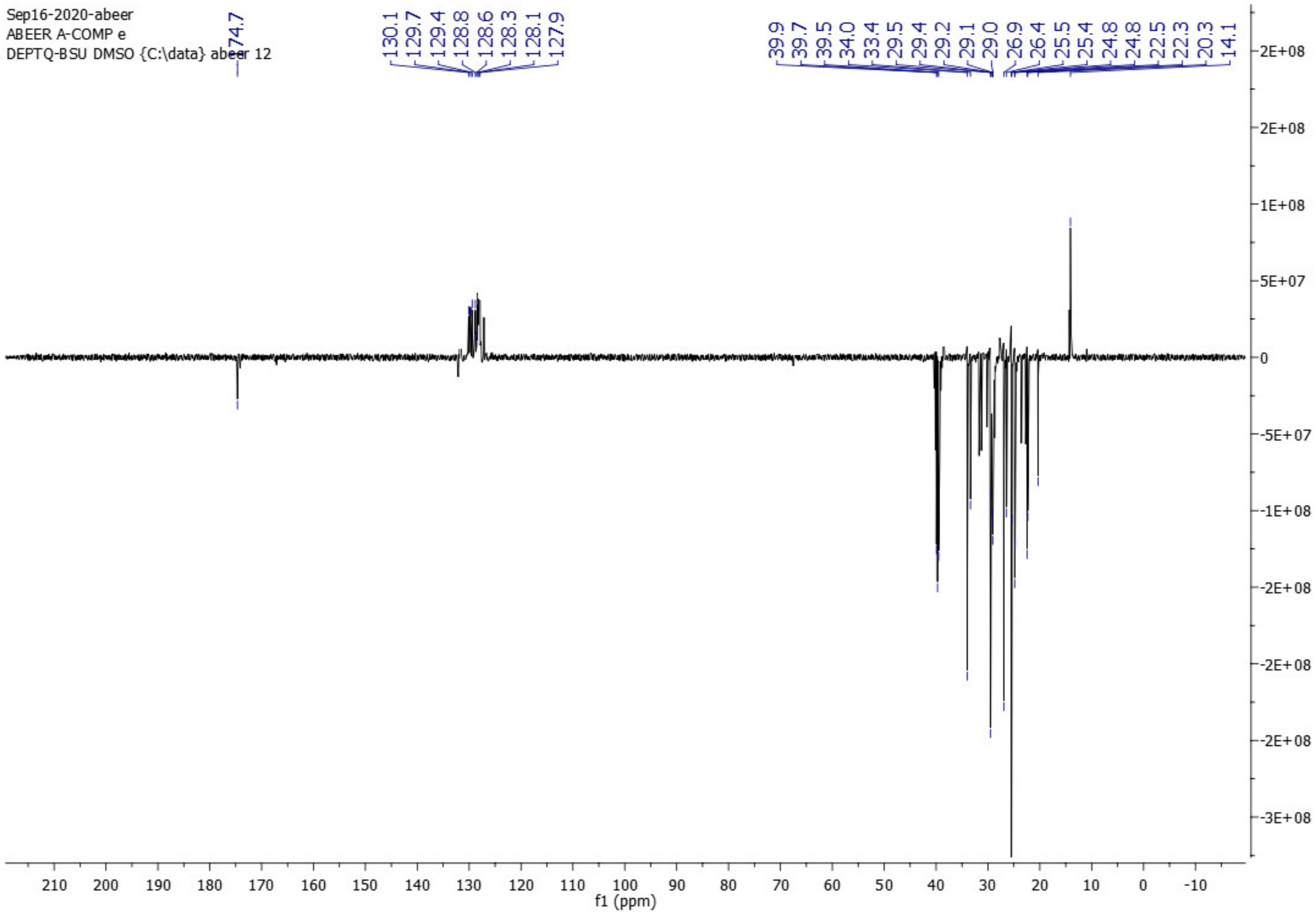

Figure S9. DEPT-Q NMR spectrum of compound **4** measured in DMSO-d_6 at 100 MHz

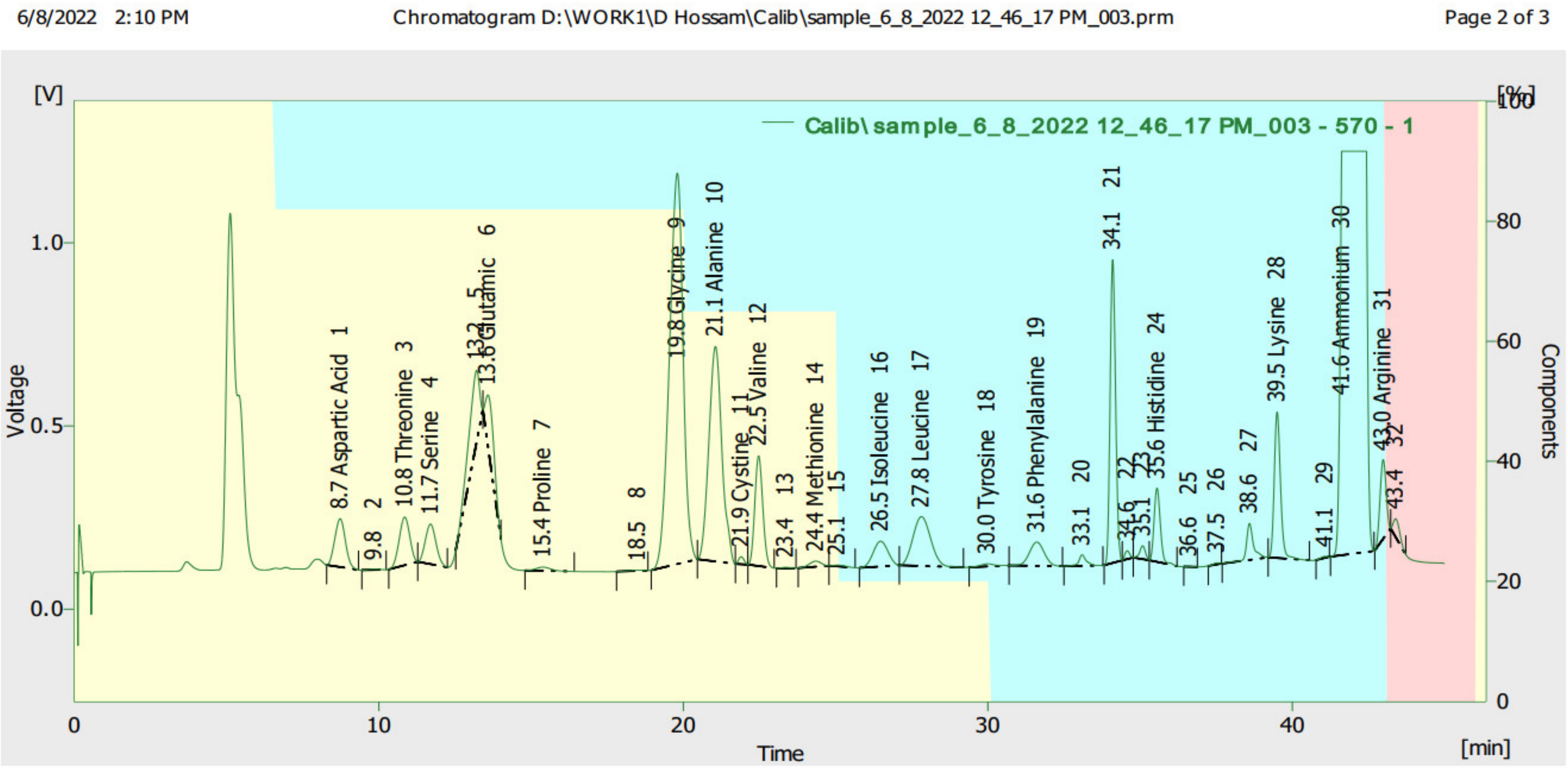

Figure S10. Amino acid sample analysis.
